# A model‐based meta analysis study of sodium glucose co‐transporter‐2 inhibitors

**DOI:** 10.1002/psp4.12934

**Published:** 2023-03-08

**Authors:** Xueting Yao, Jiawei Zhou, Ling Song, Yupeng Ren, Pei Hu, Dongyang Liu

**Affiliations:** ^1^ Drug Clinical Trial Center, Institute of Medical Innovation and Research Peking University Third Hospital Beijing China; ^2^ Center of Clinical Medical Research, Institute of Medical Innovation and Research Peking University Third Hospital Beijing China; ^3^ Division of Pharmacotherapy and Experimental Therapeutics, School of Pharmacy University of North Carolina at Chapel Hill Chapel Hill North Carolina USA; ^4^ Johnson & Johnson Pharmaceuticals (Shanghai) Ltd. Shanghai China; ^5^ Clinical Pharmacology Research Center Peking Union Medical College Hospital & Chinese Academy of Medical Sciences Beijing China

## Abstract

Type 2 diabetes mellitus (T2DM) agent sodium‐glucose co‐transporter 2 (SGLT2) inhibitors show special benefits in reducing body weight and heart failure risks. To accelerate clinical development for novel SGLT2 inhibitors, a quantitative relationship among pharmacokinetics, pharmacodynamics, and disease end points (PK/PD/end points) in healthy subjects and patients with T2DM was developed. PK/PD/end point data in published clinical studies for three globally marketed SGLT2 inhibitors (dapagliflozin, canagliflozin, and empagliflozin) were collected according to pre‐set criteria. Overall, 80 papers with 880 PK, 27 PD, 848 fasting plasma glucose (FPG), and 1219 hemoglobin A1c (HbA1c) data were collected. A two‐compartmental model with Hill's equation was utilized to capture PK/PD profiles. A novel translational biomarker, the change of urine glucose excretion (UGE) from baseline normalized by FPG (ΔUGE_c_) was identified to bridge healthy subjects and patients with T2DM with different disease statuses. ΔUGE_c_ was found to have a similar maximum increase with different half‐maximal effective concentration values of 56.6, 2310, and 841 mg/mL·h for dapagliflozin, canagliflozin, and empagliflozin respectively. ΔUGE_c_ will change FPG based on linear function. HbA1c profiles were captured by indirect response model. Additional placebo effect was also considered for both end points. The PK/ΔUGE_c_/FPG/HbA1c relationship was validated internally using diagnostic plots and visual assessment and further validated externally using the fourth globally approved same‐in‐class drug (ertugliflozin). This validated quantitative PK/PD/end point relationship offers novel insight into long‐term efficacy prediction for SGLT2 inhibitors. The novelty identified ΔUGE_c_ could make the comparison of different SGLT2 inhibitors' efficacy characteristics easier, and achieve early prediction from healthy subjects to patients.

**Study Highlights**

WHAT IS THE CURRENT KNOWLEDGE ON THE TOPIC?

Urinary glucose excretion (UGE) was used as a pharmacodynamic (PD) biomarker for sodium‐glucose co‐transporter 2 inhibitors. But the pharmacokinetic (PK)/UGE relationship was found to be different between healthy subjects and patients with type 2 diabetes mellitus (T2DM), which hindered the translation from healthy subjects to patients and the comparison of efficacy for same‐in‐class drugs.

WHAT QUESTION DID THIS STUDY ADDRESS?

We proposed a translatable PD biomarker to bridge PK/PD profiles between healthy subjects and patients with T2DM as well as to establish a quantitative relationship of biomarker/FPG/HbA1c in patients.

WHAT DOES THIS STUDY ADD TO OUR KNOWLEDGE?

A biomarker, the change of UGE from baseline corrected by corresponding fasting plasma glucose baseline (ΔUGEc), was identified to be comparable between healthy subjects and patients with T2DM. In addition, a PK/ΔUGEc/FPG/HbA1c model was developed using three SGLT2 inhibitors's data and validated by fourth SGLT2 inhibitor's data.

HOW MIGHT THIS CHANGE DRUG DISCOVERY, DEVELOPMENT, AND/OR THERAPEUTICS?

The PK/PD/end point model proposed by this study could predict end point profiles in patients for novel SGLT2 inhibitors based on PK/PD profiles in healthy subjects, which can be used to quickly determine the effective dose regimen in early phase.

## INTRODUCTION

Diabetes mellitus is a global chronic metabolize disease with the population reaching 415 million in 2015, and the number is expected to reach 642 million by 2035.^1^ Patients with type 2 diabetes mellitus (T2DM) have a significantly higher risk of cardiovascular disease, hospitalization, limb amputation, nontraumatic blindness, or liver failure.^2^ Currently, many kinds of oral long‐term hypoglycemic agents, such as metformin, sulfonylurea insulin secretagogues, thiazolidinediones, are utilized to reduce morbidity and elevate life quality for patients. However, these medications are associated with side effects, including hypoglycemia, gastrointestinal symptoms, and weight gain,^3^ and have not shown an impact on reducing cardiovascular risks so far.^4^

Sodium‐glucose co‐transporter 2 (SGLT2) inhibitors represent a novel class of oral T2DM treatment targeting at glucose transport system in the renal proximal tubule. They decrease blood glucose levels by competing with glucose for SGLT2 transporter, thus increasing kidney glucose threshold and urine glucose excretion.^5^ The novel mechanism of SGLT2 inhibitors suggested that they could be given in combination with most of the antidiabetic agents currently on the market as they shared no common pathways. Furthermore, because SGLT2 inhibition did not interact with pancreatic cells, they would not stimulate insulin release, and due to caloric loss associated with excreted glucose, thus reduce body weight.^6^ Recent research outcomes indicated that SGLT2 inhibitors can significantly decrease the morbidity caused by cardiovascular disease and hospitalization due to heart failure.^4^

Three SGLT2 inhibitors (dapagliflozin, canagliflozin, and empagliflozin) have been approved globally and more novel drugs are under development. To assist dose selection for SGLT2 inhibitors, urinary glucose excretion (UGE) was used as a pharmacodynamic (PD) biomarker in healthy subjects.^7^ The pharmacokinetic (PK)/UGE relationship was found to be different between healthy subjects and patients with T2DM, which hindered the translation of novel drug from healthy subjects to patients with T2DM and the comparison of efficacy same‐in‐class marketed drugs.^8^ Although some PK/biomarker, PK/end point, or PD/end point models for SGLT2 inhibitors have been developed, there is still a lack of studies on predicting long‐term treatment fasting plasma glucose (FPG) or hemoglobin A1c (HbA1c) profiles based on healthy subject PK data because no mechanistically translational PD biomarker was integrated to bridge PKs and end points.^9^, ^10^, ^11^, ^12^ Therefore, these models were hard to be translated for other drugs because potency and drug distribution in the target tissue of each same‐in‐class drug are different. Different PK/PD profiles and unclear PD/end points relationships increase the difficulties for the suggestion of effective dose in patients which will require a bigger sample size and longer testing duration to identify effective dosage for novel SGLT2 inhibitors in patients. Therefore, it is critical to identify a translatable PD biomarker to bridge PK/PD profiles between healthy subjects and patients with T2DM as well as to establish a quantitative relationship of biomarker/FPG/HbA1c in patients. Model‐based meta‐analysis is a knowledge‐integrated approach by summarizing numerical data in multiple kinds of literature followed by modeling these data to receive quantitative relationship of PK/PD/disease end points.^13^ It could take advantage of meta‐analysis (powerful because of fruitful studies) and modeling (ability to quantify the PK/PD/end point relationship) approaches. Theoretically, it perfectly fits the objectives of this current translational study from healthy subjects to patients.

Therefore, we first explored a system‐specific PD biomarker to establish a quantitative relationship between PK exposure and T2DM end points for SGLT2 inhibitors; then we validated this translational strategy using the fourth marketed same‐in‐class SGLT2 inhibitor (ertugliflozin).

## MATERIALS AND METHODS

### Overall study strategy

The overall study strategy is shown in Figure S1. Briefly, we first did a population PK analysis for each studied drug. Then we proposed a mechanistic and translatable PD biomarker, ΔUGE corrected by corresponding FPG baseline (ΔUGE_c_). This new biomarker exhibited a similar PK/ΔUGE_c_ relationship for the same‐in‐class drug PDs and a consistent PK/ΔUGE_c_ relationship between healthy subjects and patients with T2DM. Furthermore, we used ΔUGE_c_ to develop the PK/ΔUGE_c_/FPG/HbA1c relationship sequentially followed by internal and external validation.

### Search strategies and data collection

Initial English literature research on the PubMed database was conducted for all SGLT2 inhibitors up to July 2016 by searching terms of drug name and “Diabetes Mellitus,” and filtering with “Clinical Trial.” Articles about the disease other than type 2 diabetes were excluded. Studies of patients with moderate or severe kidney impairment or hepatic insufficiency were also excluded in end point data. Studies with one of the following condition were selected: (1) containing clinical PK data (sampling time, plasma drug concentrations, and dose regimens of both healthy subjects and patients); (2) containing clinical PD (dose, change from baseline UGE values with corresponding FPG of both healthy and T2DM subjects) data from studies without other antihyperglycemic medications; (3) containing clinical FPG and HbA1c data (including FPG and HbA1c with corresponding baseline values, dose, and therapy category) from trials without insulin treatment. Selected study papers are shown in the supplementary citation list. The plasma concentrations of each drug, the UGE data in 24 h with corresponding FPG, and clinical outcomes containing time course of FPG and HbA1c in both placebo and drug groups meeting pre‐set criteria were collected. All the data were extracted from tables directly or graphics with Digitizer (Graph Digitizer version 19). Other information, including authors, journal name, publication time, and clinical information, like patient number, time since diagnosis, body height and weight, and fed or fasted state, were also collected.

### Model development

**FIGURE 1:**
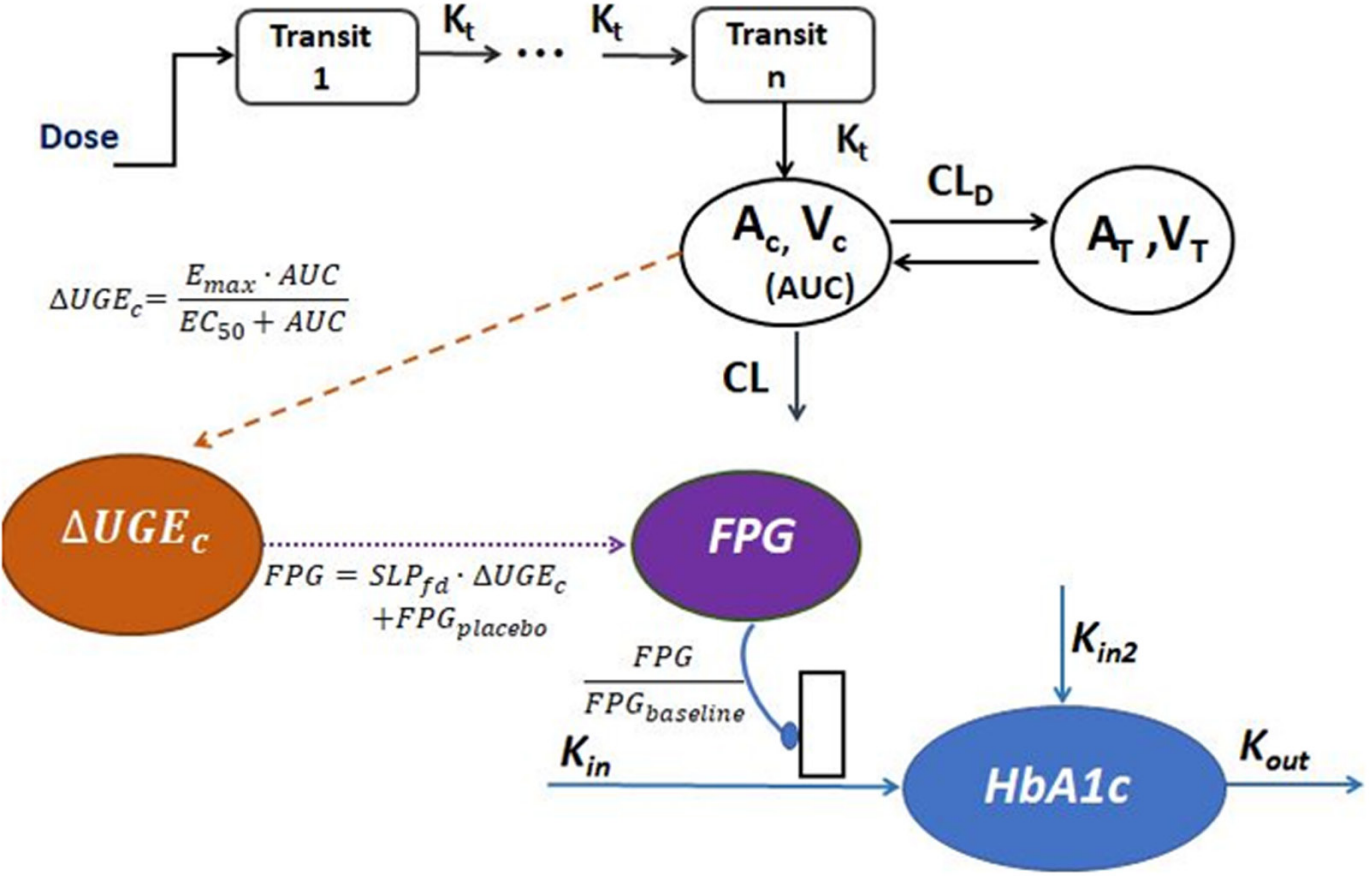
The proposed model structures for PK, PK/PD, and PD/end points. AUC, area under the concentration‐time curve; CL, clearance; EC_50_, half‐maximal effective concentration; E_max_, maximum effect; FPG, fasting plasma glucose; *K*_in_, increase rate constant; *K*_out_, elimination rate constant; PD, pharmacodynamic; PK, pharmacokinetic.

The PK/PD/end point relationships were constructed using nonlinear mixed‐effects (NONMEM) modeling approach on NONMEM (version 7.2) interfaced with PSN (version 4.2.0).^14^, ^15^ Fitting was conducted with first‐order conditional estimation with interaction method. R software (version 3.0.2) in R studio (version 0.97.551) was used for modeling‐ready dataset creation and generation of plots. The schematic model structure was shown in Figure 1. To accommodate the different population sizes in meta‐studies, we used the square root of sample size in each study as the weighting of data. Different study designs and populations were considered as covariates, such as fed or fasted state, and tested in model development. According to treatment history and therapy regimens, patients were divided into different treatment types: naïve therapy (patients naïve to oral hypoglycemic agents), non‐naïve therapy (patients had taken hypoglycemic agents but had undergone a more than 2‐week washout period), add‐on therapy (patients were in combination treatment in the study), and mixed (contained both naïve and non‐naïve therapy and unknown treatment type).

#### PK model

Data from all collected PK studies were pooled to form an integrated dataset for each drug. A two‐compartmental model with first order elimination and transit absorption function was established to predict drug exposure for three SGLT2 inhibitors. As the fed state prolonged the time to maximum concentration of dapagliflozin,^16^ the fed or fasted state was screened to identify the effect on the absorption rate constant. The details for PK model development are supplied in Appendix S1.

#### PK/PD model

Drug exposure in patients with T2DM expressed as area under the concentration‐time curve (AUC) was simulated using the established population PK model for each agent. AUC at steady status was selected to drive PD changes. Change of UGE from baseline corrected by corresponding baseline FPG was characterized as translational drug efficacy biomarker (ΔUGE_c_) based on pharmacological mechanism (Equation 1). An empirical maximum effect (E_max_) model was utilized to demonstrate drug exposure‐biomarker dynamics as shown as Equation 2.

(1) $$∆{\mathrm{UGE}}_{c}=\left(\mathrm{UGE}-{\mathrm{UGE}}_{\text{baseline}}\right)/{\mathrm{FPG}}_{\text{baseline}}$$

(2) $$∆{\mathrm{UGE}}_{c}=\frac{E_{\max}.{\mathrm{AUC}}_{0-24h}}{{\mathrm{EC}}_{50}+{\mathrm{AUC}}_{0-24h}}$$

Where ΔUGE_c_ is the delta 24‐h UGE (change from baseline) corrected by baseline FPG, reflecting a 24 h glucose clearance. E_max_ is the maximal drug response. EC_50_ is the exposure producing half‐maximal drug response. AUC_0–24 h_ is the area under the concentration‐time curve for SGLT2 inhibitors at steady status within a 24‐h interval.

#### PD/end points model

Because these three SGLT2 inhibitors have the same target and the following biological signaling pathway from target to the end point is the same, their FPG and HbA1c data were pooled together to explore the PD/end point quantitative relationship universal for all drugs in this class.

The placebo and drug effects on FPG are described in Equations 3 and 4, respectively.

(3) $$\begin{array}{c} \;{\mathrm{FPG}}_{\text{placebo}}={\mathrm{FPG}}_{\text{baseline}}+P_{\text{fmax}}.\left(1-e^{-K_{\mathrm{fp}}.t}\right)+{\mathrm{DIS}}_{\mathrm{fp}}.t \end{array}$$

(4) $$\begin{array}{c} \;\mathrm{FPG}={\mathrm{FPG}}_{\text{placebo}}+{\text{SLOPE}}_{\mathrm{fd}}.∆{\mathrm{UGE}}_{c} \end{array}$$

where P_fmax_ is the maximal placebo effect on FPG under placebo treatment. DIS_fp_ is a linear coefficient on time describing disease progression on FPG. *K*_fp_ is the first‐order rate constant of FPG under placebo treatment. SLOPE_fd_ is the drug effect for FPG associated with ΔUGE_c_. An exponential function was introduced to describe the change of PFG by placebo effect, and a liner function was introduced to describe the slow change of FPG along with disease progression.

Both placebo and drug effects' models on the HbA1c‐time course were established. The placebo model of HbA1c was similar to FPG and is shown in Equation 5.

(5) $$\begin{array}{c} \mathrm{HbA}1c_{\text{placebo}}=\mathrm{HbA}1c_{\text{baseline}}-P_{\text{hmax}}.\left(1-e^{-K_{\mathrm{hp}}.t}\right)+{\mathrm{DIS}}_{\mathrm{hp}}.t \end{array}$$

where P_hmax_ is the maximal decreasing extent of HbA1c under placebo treatment, *K*_hp_ is the first‐order rate constant of HbA1c under placebo treatment. DIS_hp_ is a linear coefficient on time describing disease progression on HbA1c.

The drug effects on the HbA1c model are shown in Equations (6), (7), (8).

(6) $$K_{\text{in}}=K_{\text{out}}.\mathrm{HbA}1c_{\text{baseline}}-K_{\text{in}2}$$

(7) $$\begin{array}{c} \frac{d\left(\mathrm{HbA}1c_{\text{drug}}\right)}{dt}=\frac{\mathrm{FPG}}{{\mathrm{FPG}}_{\text{baseline}}}\cdot K_{\text{in}}\\ \;+K_{\text{in}2}-K_{\text{out}}\cdot \mathrm{HbA}1c,\mathrm{HbA}1c_{\text{drug}}\left(0\right)=0 \end{array}$$

(8) $$\mathrm{HbA}1c=\mathrm{HbA}1c_{\text{placebo}}+\mathrm{HbA}1c_{\text{drug}}$$

Where *K*_in_ is the FPG‐dependent increase rate constant of HbA1c, and *K*_in2_ is the FPG‐independent increase rate constant of HbA1c, and *K*_out_ is the elimination rate constant of HbA1c. Because both FPG and postprandial plasma glucose (PPG) were highly contributed to HbA1c, a zero‐order rate constant (*K*_in2_) was introduced to stand for other factors that increase HbA1c except for FPG. Additive inter‐study variability was proposed for P_fmax_ and P_hmax_. In addition, inter‐study variability of other parameters in both of FPG and HbA1c models were expressed as exponential form as assumed to be log‐normally distribution. The residual variability was described using proportional or combined proportional and additive error models weighted by the square root of sample size in the PK/PD/end point analysis. Covariates including subject type (healthy vs. T2DM), age, gender, body weight, fed or fasted state were tested in population PK and PD model selection. Covariates, including washout period, treatment type, study design, were tested in the PD/end point model for all three drugs. We only considered the covariates that had been measured in all the studies included in our dataset for all three drugs.

### Model evaluation

The final models were assessed by objective function value (OFV), the precision of parameter estimates (relative standard error of the estimates), diagnostic plots (population predictions vs. observations, individual predictions vs. observations, and conditional weighted residuals vs. population predictions or time), and visual predictive checks (VPCs).

### External validation

The final PK/PD/end points model was validated using the external data from ertugliflozin (PF‐04971729), a fourth SGLT2 inhibitor that was approved by the US Food and Drug Administration (FDA) at the end of 2017. Data of ertugliflozin from healthy subjects and patients with T2DM with normal renal function were used to develop the PK/PD model for ertugliflozin followed by prediction of HbA1c profiles in patients with T2DM. HbA1c‐time profiles from three phase III studies were utilized to confirm the prediction results. Predicted reductions in HbA1c were compared with the observations to validate the prediction performance of the PK/PD/end points model with novelly proposed biomarker ΔUGEc.

## RESULTS

### Data summary

Three SGLT2 inhibitors (dapagliflozin, canagliflozin, and empagliflozin) were collected in this analysis due to the preset criteria in 2016. The primary search results contained 145 articles of potential interest, among which 34 for dapagliflozin, 23 for canagliflozin, and 23 for empagliflozin satisfied pre‐set criteria as shown in Figure S2. A total of 880 summary‐level drug concentrations, 27 summary‐level ΔUGE_c_, 848 (195 in placebo and 653 in drug) summary‐level FPG data points, and 1219 (290 in placebo and 929 in drug) summary‐level HbA1c data points were extracted from these studies. The dose ranges in PK data covered dose ranges in PD and end points data except for 1 mg dose in dapagliflozin. UGE, FPG, and HbA1c data in each arm had its corresponding baseline value. This analysis included the studies in different designs and in both healthy subjects and patients. The included studies and mean demographic information are shown in Tables S1 and S2. We also included 26 HbA1c data points from ertugliflozin for external validation.

### PK/PD model

The two‐compartmental models with transit compartments well‐described the time course of concentrations of three drugs. The PK model parameter estimates are shown in Table 1. Fed or fasted state was added as a covariate of absorption rate constant for dapagliflozin because food showed significant effects on absorption. The AUC in 24 h corresponding to the UGE data were estimated from the PK model and the steady‐state AUC in 24 h after long‐term treatment was 51.39 ng/mL·h for dapagliflozin, 83.6 ng/mL·h for canagliflozin, and 235.3 ng/mL·h for empagliflozin.

**TABLE 1:** PK model parameter estimates.

Parameters (unit)	Definition	Estimates	RSE (%)	IIV (%)
*PK model of dapagliflozin*				
CL (L/h)	Clearance from central compartment	19.5	4.00	2.50
Vc (L)	Volume of distribution in central compartment	82.0	6.90	9.44
CL_D_	Distribution clearance between central and peripheral compartment	10.3	7.10	–
V_T_ (L)	Volume of distribution in peripheral compartment	122	9.60	5.83
*K*_*t*_ (h^−1^)	Absorption rate constant between transit compartments	6.50	12.4	23.0
Fed	Covariate of fed or fasted state	0.254	29.8	–
σ^2^_pro_	Variance of proportional residual error in PK model	0.457	–	–
σ^2^_add_	Variance of additive residual error in PK model	0.462	–	–
*PK model of canagliflozin*				
CL (L/h)	Clearance from central compartment	12.0	6.20	5.90
Vc (L)	Volume of distribution in central compartment	85.5	4.60	2.43
CL_D_	Distribution clearance between central and peripheral compartment	9.77	10.8	12.8
V_T_ (L)	Volume of distribution in peripheral compartment	108	6.40	3.14
*K*_*t*_ (h^−1^)	Absorption rate constant between transit compartments	6.38	4.60	3.17
σ^2^_pro_	Variance of proportional residual error in PK model	0.126	–	–
*PK model of empagliflozin*				
CL (L/h)	Clearance from central compartment	4.25	6.10	5.69
Vc (L)	Volume of distribution in central compartment	30.6	9.50	5.63
CL_D_	Distribution clearance between central and peripheral compartment	1.37	13.5	–
V_T_ (L)	Volume of distribution in peripheral compartment	28.3	26.5	22.0
*K*_*t*_ (h^−1^)	Absorption rate constant between transit compartments	4.13	6.90	5.67
σ^2^_pro_	Variance of proportional residual error in PK model	0.521	–	–

Abbreviations: IIV, interindividual variability; PK, pharmacokinetic.

**FIGURE 2:**
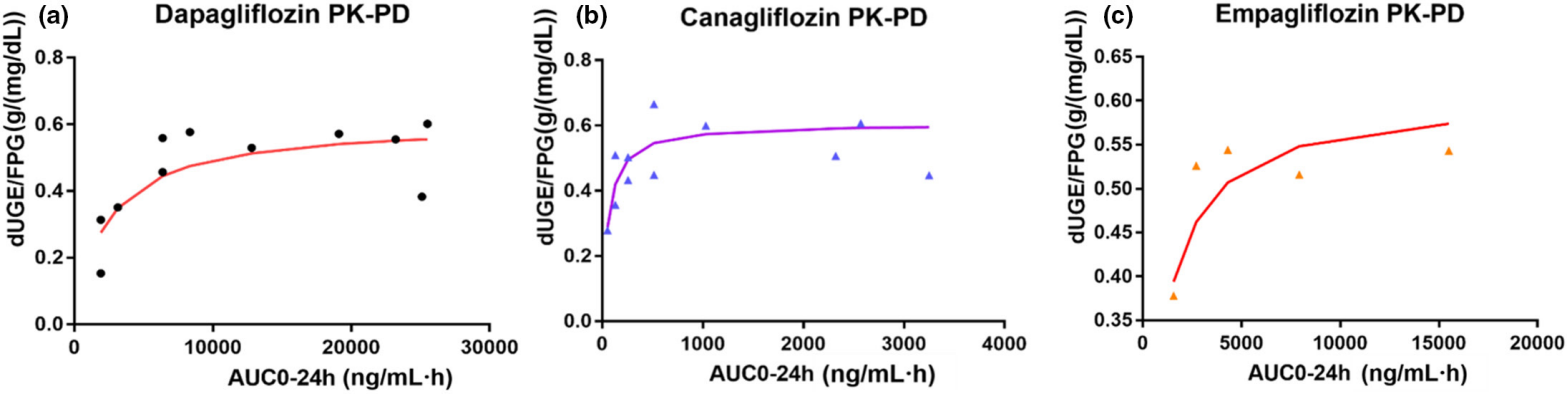
The relationships between drug exposure and ΔUGEc of dapagliflozin (a), canagliflozin (b), and empagliflozin (c). The line is linked by the model predictions, whereas the dots are the observations. AUC_0–24 h_, area under the concentration‐time curve for SGLT2 inhibitors at steady status within a 24‐h interval; FPG, fasting plasma glucose; PD, pharmacodynamic; PK, pharmacokinetic; UGE, urine glucose excretion.

The observed relationships between drug exposure and ΔUGE_c_ for three drugs are shown in Figure 2. The PK/PD model parameter estimates are shown in Table 2. In the final PK/PD model, exposure‐ΔUGE_c_ relationships of the three SGLT2 inhibitors shared the same E_max_ of 0.606 g/(mg/dL), because the data from the three drugs showed similar trends in inhibition of ΔUGE_c_. The EC_50_ estimates were 56.6, 2310, and 841 ng/mL·h for dapagliflozin, canagliflozin, and empagliflozin, respectively.

**TABLE 2:** PK/PD and PD/end point model parameter estimates.

Parameters (unit)	Definition	Estimates	RSE (%)	IIV (%)
*PK/PD model*				
E_max_ (g/(mg/dL))	Maximal drug efficacy	0.606	4.40	–
Dapa‐EC_50_ (ng/mL·h)	AUC resulting half maximal effect for dapagliflozin	56.6	27.1	–
Cana‐EC_50_ (ng/mL·h)	AUC resulting half maximal effect for canagliflozin	2310	23.3	–
Empa‐EC_50_ (ng/mL·h)	AUC resulting half maximal effect for empagliflozin	841	30.4	–
σ^2^_pro_	Variance of proportional residual error in PK/PD model	0.222	–	–
σ^2^_add_	Variance of additive residual error in PK/PD model	0.0646	–	–
*PD/end point model*				
FPG_baseline_ (mg/dL)	The estimated population FPG baseline level	160	1.00	5.40
P_fmax1_ (mg/dL)	Maximal placebo effects on FPG in naïve group	1.45	26.0	5.74
P_fmax2_ (mg/dL)	Maximal placebo effects on FPG in non‐naïve group	1.90	111	–
P_fmax3_ (mg/dL)	Maximal placebo effects on FPG in add‐on group	−1.37	74.0	6.83
P_fmax4_ (mg/dL)	Maximal placebo effects on FPG in mixed group	4.30	55.0	0.87
*K*_fp_ (week^−1^)	FPG rate constant of placebo effect	0.340	55.0	42.7
DIS_fp_ (mg/dl/100 weeks)	Disease progression rate of FPG	3.13	41.0	–
SLOPE_fd_ (mg/dL^2^)	FPG decrease rate by drug effects	−43.3	4.10	29.2
σ^2^_add, FPG_	Variance of residual error of FPG	0.0330	–	–
HbA1c_baseline_ (%)	The estimated population HbA1c baseline level	7.92	1.00	3.90
P_hmax1_ (%)	Maximal placebo effects on HbA1c in naïve group	−0.200	17.0	0.08
P_hmax2_ (%)	Maximal placebo effects on HbA1c in non‐naïve group	0.0510	109	–
P_hmax3_ (%)	Maximal placebo effects on HbA1c in add‐on group	−0.230	16.0	0.13
P_hmax4_ (%)	Maximal placebo effects on HbA1c in mixed group	−0.06	81.0	0.20
*K*_hp_ (week^−1^)	HbA1c rate constant of placebo effect	0.240	21.0	29.4
DIS_hp_ (%/100 weeks)	Disease progression rate of HbA1c	0.310	33.0	136.7
*K*_out_ (week^−1^)	Decrease rate of HbA1c	0.200	3.00	16.4
*K*_in2_ (%/week)	Increase rate of HbA1c independent of FPG	0.500	5.00	–
σ^2^_add, HbA1c_	Variance of residual error of HbA1c in PD/FPG/HbA1c model	0.006	–	–

Abbreviations: FPG, fasting plasma glucose; IIV, interindividual variability; PD, pharmacodynamic; PK, pharmacokinetic.

### PD/end point model

The placebo effect models indicated patients with different treatment types tended to have different end point (HbA1c and FPG) time courses in the placebo group. The placebo model parameters were estimated for the four types of the patient group, respectively. An exponential function adequately described the change in placebo FPG response over time with *K*_fp_ of 0.340 week^−1^ or a half‐life of 2 weeks. The rate of disease progression in FPG was estimated with DIS_fp_ of 3.13 mg/dL increase in 100 weeks. In the final placebo model of FPG, the maximum difference under placebo in naïve group (P_fmax1_), non‐naïve groups (P_fmax2_), add‐on group (P_fmax3_), and a mixed group (P_fmax4_) were 1.45 mg/dL, 1.90 mg/dL, −1.37 mg/dL, and 4.30 mg/dL, respectively. Most patients had FPG increase under placebo treatment, except for the add‐on group, where patients got their FPG under control with the help of other agents in combination. The drug efficacy on FPG is estimated by SLOPE_fd_ of −43.3 mg/dL,^2^ which made the prediction of long‐term FPG changes by ΔUGE_c_ for all three drugs possible. In the HbA1c placebo model, the estimated P_hmax_ value in HbA1c for naïve groups (P_hmax1_), add‐on groups (P_hmax3_), and mixed groups (P_hmax4_) were −0.20%, −0.23%, and −0.06% decrease from baseline while that of non‐naïve groups (P_hmax2_) was 0.051% increase from baseline. In the drug efficacy model, the degeneration rate constant of HbA1c with *K*_out_ of 0.20 week^−1^ and FPG‐independent increase rate constant of HbA1c with *K*_in2_ of 0.50%/week were estimated. Treatment type is not significant on other parameters here. The other information of parameter estimates for PD/end point models could be found in Table 2.

### Model evaluation

**FIGURE 3:**
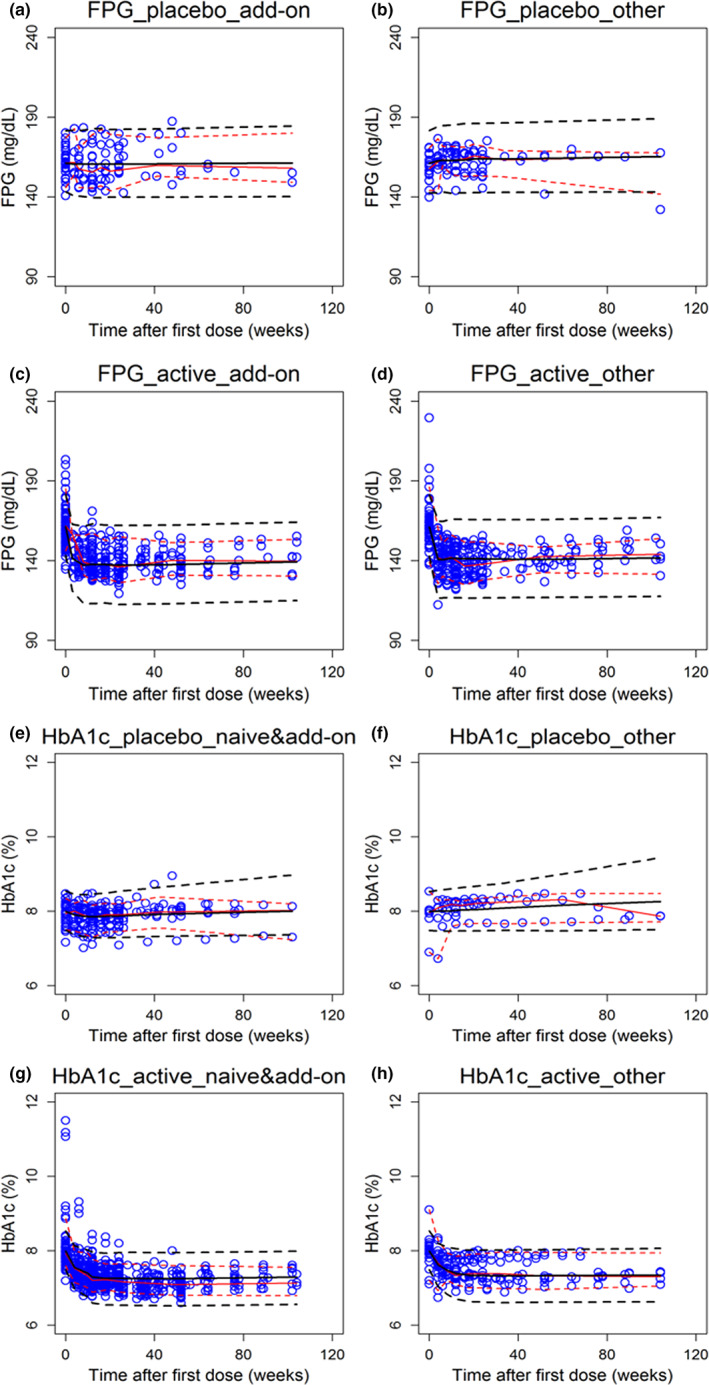
VPC plots in add‐on group (a), and naïve, non‐naïve, mixed groups (b) of placebo effects of FPG model across time; in add‐on group (c), and naïve, non‐naïve, mixed groups (d) of drug effects of FPG model across time. VPC plots in naïve and add‐on groups (c), non‐naïve, mixed groups (d) of the placebo effects of HbA1c time course model; and drug effects model of HbA1c changes in naïve and add‐on groups (g) and in non‐naïve, mixed groups (h). The circles are the observations and the red solid line is the median and the red dashed lines are the 5% and 95% percentile of the observations. The black solid line is the median and the black dashed lines are the 5% and 95% percentile of simulations. FPG, fasting plasma glucose; VPC, visual predictive check.

All the final models showed good agreement and adequate accuracy. The diagnostic plots of PK and PK/PD models are shown in Figures S6 and S7, respectively. The diagnostic plots and VPC plots of end point models are shown in Figure S8 and Figure 3. No systematical biases were inspected in these plots in both FPG and HbA1c models and in both placebo and drug groups. Overall, the model described the central tendency of the PD/end point profiles in the population level as most of the observed median falls within the simulated 95% predictive interval for the median.

### External validation

**FIGURE 4:**
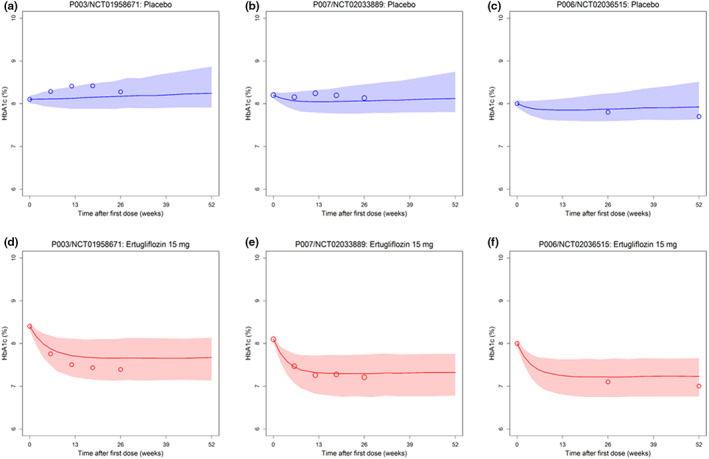
Prediction and observation for the change over time of HbA1c in the placebo group (a, *n* = 153, b, *n* = 209, and c, *n* = 153) and in T2DM subjects with monotherapy of ertugliflozin 15 mg (d, *n* = 151), T2DM subjects administration ertugliflozin 15 mg and add‐on to metformin (e, *n* = 151), and T2DM subjects administration ertugliflozin 15 mg and add‐on to metformin and sitagliptin (f, *n* = 151) from three studies. The circles are the observations and the shadows are 90% prediction interval.

Ertugliflozin HbA1c change over time from three published clinical trials was collected for external validation. The end points in two types of patient groups (naïve and non‐naïve with monotherapy and add‐on therapy with metformin or sitagliptin) were predicted by the PD/end point model and compared with the observations. The validation results are shown in Figure 4. Most of the observations fell inside the 90% prediction interval, which indicated the disease end points could be accurately estimated.

## DISCUSSION

Generally, we could establish a PK/PD relationship for the new drugs in healthy subjects and PK/PD/end point relationships for marketed same‐in‐class drugs in patients. The marketed drug PD/end point relationships could be borrowed in new drug development because they are system‐specific rather than drug‐specific.^17^ Therefore if a PK/PD relationship was suggested to be similar between healthy subjects and patients with T2DM, we can predict end point profiles in patients for novel drug based on PK/PD profiles in healthy subjects and PD/end point relationships from marketed drugs. Gibbs et al.^18^ did a great case study using weighted average inhibition of DPP‐4 enzyme (biomarker) to translate PK/PD profiles of DPP‐4 inhibitors to PD/end point profiles. We established a PK/PD/endpoints model to quantitatively predict FPG and HbA1c changes in patients based on PK/PD profiles in the early phase. Here, we collected PK/PD/end points data followed by a population PK analysis for each studied drug. Considering the critical role of PD biomarker in early phase drug development, we proposed a mechanistic PD biomarker, ΔUGE_c_, which bridged PK exposure and disease end points (FPG and HbA1c). Considering different disease progression led by different types of patients and trial designs, ΔUGE_c_‐FPG‐HbA1c relationships were constructed for four categories (naïve, non‐naïve, add‐on, and mixed). The model was also validated using ertugliflozin data from published clinical studies. External validation result demonstrated good predictability for disease endpoint by this model, and confirmed the PD/HbA1c relationship proposed in this paper. This PK/PD/end point model lays a solid foundation for facilitating drug development for SGLT2 inhibitors.

In this analysis, we built PK, PK/PD, and PD/end point models sequentially because the data used in three parts were from different studies and different subjects. The population PK profiles of dapagliflozin, canagliflozin, and empagliflozin in patients and healthy people were similar and described by a two‐compartment model with first‐order absorption, respectively. Transit compartments showed better goodness‐of‐fit than the un‐physiological lag time model in describing absorption delay. Two transit compartments were applied for dapagliflozin and empagliflozin and four transit compartments for canagliflozin, indicating three drugs varied in drug formulation and physicochemical properties.^19^ The values of clearance estimates for three drugs were close to previous studies.^20^, ^21^, ^22^ No significant food effects were previously found for any of the three drugs exposure,^23^, ^24^, ^25^ but we found that food could significantly influence the *K*_*t*_ parameter (*p* < 0.005) in dapagliflozin, which is consistent with a longer time to maximum concentration of dapagliflozin under fed condition.^26^

Mechanism‐based PK/PD model with system‐dependent parameters is critical and useful, especially in the early phase of new drug development, and could be translated to other same‐in‐class drugs. If a PD biomarker could be detectable in healthy subjects and be predictive of end points in patients, early PD profiles of the novel drug in healthy subjects could be utilized to predict efficient dosage in patients after long‐duration treatment with aid of a system‐dependent model. We initially used ΔUGE as a PD biomarker because of its mechanism of action and we found that the maximum effect and affinity on UGE was significantly different between different SGLT2 inhibitors and between patients and healthy subjects. It was consistent with one paper which even concluded that the ΔUGE has no relationship with FPG or HbA1c, which impeded the achievement of our preset aims.^27^ Therefore, we proposed a better mechanistic PD biomarker to construct this system‐dependent model. According to the mechanism of action for UGE production and SGLT2 inhibitors, previous research obtained ΔUGE from a function of glucose filtration rate (GFR), plasma glucose (PG), drug concentration (C), drug efficacy (I_max_, half‐maximal inhibitory concentration [IC_50_]), and re‐absorption fraction (f_reabs_).

(9) $$\begin{array}{c} \frac{\text{dUGE}}{dt}=\mathrm{GFR}.\mathrm{PG}-f_{\text{reabs}}.\mathrm{GFR}.\mathrm{PG}.\left(1-\frac{I_{\max}.C}{{\mathrm{IC}}_{50}+C}\right) \end{array}$$

Therefore, GFR and FPG corrected ΔUGE could better describe drug effect than ΔUGE in theoretically. Because AUC of plasma glucose over 24 h (AUEC_0–24h_) represents plasma glucose exposure over the whole duration, AUEC_0–24h_ or mean plasma glucose (MPG) could best bridge PK and disease end points. However, it is hard to receive multiple plasma glucose levels on 1 day in late‐phase clinical trials. We compared the relationship between PK/ΔUGEc corrected by FPG and PK/ΔUGEc corrected by MPG using the modeling method based on our limited data, ΔUGEc corrected by FPG showed a better relationship (Figure S4) suggested by smaller OFV value (−57.265 vs. −61.607). Additionally, to avoid the bias caused by impaired kidney and hepatic function, the studies in patients with kidney or liver impairment were excluded (only 3 studies provided such data). Because FPG data are easier to acquire than AUEC_0–24h_ or MPG, the PK/PD/end point model using ΔUGEc corrected by FPG will exhibit more general applications in predicting PD/end point profiles in patients. Finally, we used ΔUGEc as a valid PD biomarker to bridge PK exposure and disease end points. The results showed that the newly proposed PD biomarker was acceptable to fulfill its mission.

The PK/PD model showed that three drugs with the same E_max_ of 0.606 g/(mg/dL), which was similar to the estimated I_max_ value of 0.35^9^ when fixed GFR by 125 mL/min (normal kidney function). We assumed that the E_max_ is similar in healthy subjects and patients with T2DM, which was also suggested by the plot of two studies PK/ΔUGEc data from canagliflozin and dapagliflozin.

In the end point models, we first analyzed the changes of FPG and built placebo and drug effects on FPG sequentially. The drug efficacy on FPG decrease was associated with ΔUGE_c_ in a linear relationship, which is consistent with its mechanism. Then the HbA1c changes over time were described as dependent on FPG changes and also composed of both placebo and drug effects. The mean FPG (160 mg/dL) and HbA1c (7.92%) baseline in patients with different treatment types were similar and close to previous studies because the clinical trials included in our study shared similar patient acceptance criteria.^28^ The maximal placebo effect on FPG was found to be different in patients with different treatment types. Patients in add‐on treatment had significantly decreased maximum placebo effects on FPG compared with other groups (1.37 mg/dL decrease from FPG baseline vs. 1.45–4.30 mg/dL increase from FPG baseline), mainly because of the confounding lowering FPG effect from the combined hypoglycemic drugs. Because naïve patients usually had shorter time with T2DM and were better controlled by diet and exercise, the placebo effects on HbA1c in these patients were similar to patients who were still on another hypoglycemic drug (add‐on patients; 0.20% decrease from HbA1c baseline vs. 0.23% decrease from HbA1c baseline). Whereas the placebo effects on HbA1c in the non‐naïve group increased a little (0.051%) from HbA1c baseline, which suggested that diet and exercise may not work well enough for non‐naïve T2DM and patients should also take antidiabetic drugs to achieve a decrease in HbA1c.^29^ The disease progression was estimated to be a 0.16% increase in HbA1c per year, which was consistent with previous studies on 0.2% nature disease progression in HbA1c.^29^ The add‐on patients sharing the same disease progression with other groups indicated that non‐insulin hypoglycemic agents did not appear to slow this progression significantly. The FPG responses in drug effects with a *K*_fp_ of 0.34 weeks^−1^ or a half‐life of 2 weeks indicated a 2‐week continuous treatment could show a significant decrease in FPG. It was noted that this decrease rate of FPG was faster than that of HbA1c as the elimination constant rate of HbA1c (*K*_out_) was estimated to be 0.20 weeks^−1^ or a half‐life of 3.5 weeks. This was consistent with previous studies that HbA1c usually took a long time to reach steady‐state than FPG and could be explained by a 3‐month delay that has been agreed clinically.^29^ Furthermore, both FPG and PPG were highly contributed to HbA1c.^30^ The relative contribution of PPG decreased from lowest HbA1c to highest HbA1c, whereas the contribution of FPG increased.^31^ To better fit the FPG‐dependent HbA1c time course model, an FPG‐independent increase rate constant *K*_in2_ of 0.50% per week was included. This zero‐order rate constant stood for all other factors that increase HbA1c except for FPG.^32^ Other reported methods, such as setting an HbA1c lower limit or changing the weight of FPG on HbA1c, could also help interpret the data differently.^28^, ^31^ Considering both blood glucose and hemoglobin contributed to HbA1c, and the FPG is not representative of overall blood glucose, a zero‐order rate constant (*K*_in2_) of HbA1c generation was added to attempt to account for HbA1c increase of other cause. We finally selected the model with the lowest OFV (OFV values −573.68) and most understandable in the mechanism. Further studies should be done to investigate the inconsistency of the two rates. However, in the PD/end point model, the effect of other hypoglycemic drugs used in combination on FPG or HbA1c in add‐on group patients cannot be isolated from placebo effect, which is one of the limitations of this study. This study proposed a PK/ΔUGE_c_/FPG/HbA1c model for bridging PK/PD/end point profiles between the healthy population and the T2DM patient population based on the assumption of PK and PD/end points similarity between these two populations. The PK/PD data were collected from patients with glomerular filtration rate more than 60 ml/min/1.73 m^2^, this assumption is more reliable when patients with T2DM have normal or at least mild injured renal function because there is an interaction between renal function and SGLT2 inhibitors.^33^ This model might be more useful in predicting patients with normal or mild injured renal function, which is also one of the limitations of this study. The similarity of PD/end point relationships between healthy subjects and patients with T2DM with moderate to severe renal injury are still needed to be verified using more clinical data.

In conclusion, we developed a PK/ΔUGE_c_/FPG/HbA1c model using data from three SGLT2 inhibitors and then used the PD data from ertugliflozin to validate the PD/end point model. External validation result demonstrated good predictability for disease end point by this model and confirmed the PD/HbA1c relationship proposed in this paper. This PK/PD/end point model may lay a solid foundation for facilitating drug development for SGLT2 inhibitors.

## AUTHOR CONTRIBUTIONS

X.Y. wrote the manuscript. P.H. and D.L. designed the research. Y.R. and L.S. performed the research. X.Y. and J.Z. analyzed the data.

## FUNDING INFORMATION

The study was supported by the “13th Five‐Year” National Major New Drug Projects (No. 2017ZX09101001‐002‐001), Capital's Funds for Health Improvement, and Research (CFH 2022‐2Z‐40917) and Bill & Melinda Gates Foundation (INV‐007625).

## CONFLICT OF INTEREST STATEMENT

The authors declared no competing interests for this work.

## Supporting information


Appendix S1
