# Supplementary material for: A model‐based meta analysis study of sodium glucose co‐transporter‐2 inhibitors
Source: CPT Pharmacometrics Syst Pharmacol. 2023 Mar 8;12(4):487–99. doi: 10.1002/psp4.12934 (PMC10088079; doi:10.1002/psp4.12934)
Supplement: Supplementary file 1 — Appendix S1 [file PSP4-12-487-s001.docx]

**1. Supplementary Methods**

1.1 PK base model

A two-transit compartment absorption and two-compartment distribution with first order elimination model was established for dapagliflozin and empagliflozin to predict drug exposure. Four-transit compartment was selected to best describe the absorption delay of canagliflozin.

PK base model of dapagliflozin and empagliflozin:

$\frac{dA_{D}}{dt}=-K_{t}\cdot A_{D}$ (Equation S1)

$\frac{dA1}{dt}=K_{t}\cdot A_{D}-K_{t}\cdot A1$ (Equation S2)

$\frac{dA2}{dt}=K_{t}\cdot A1-K_{t}\cdot A2$ (Equation S3)

$\frac{dA_{C}}{dt}=K_{t}\cdot A2-\frac{CL}{Vc}\cdot A_{C}-\frac{{CL}_{D}}{V_{C}}\cdot A_{C}+\frac{{CL}_{D}}{Vt}\cdot A_{T}$ (Equation S4)

$\frac{dA_{T}}{dt}=\frac{{CL}_{D}}{V_{C}}\cdot A_{C}-\frac{{CL}_{D}}{Vt}\cdot A_{T}$ (Equation S5)

PK base model of canagliflozin:

$\frac{dA_{DCan}}{dt}=-K_{tCan}\cdot A_{DCan}$ (Equation S6)

$\frac{dA_{1Can}}{dt}=K_{tCan}\cdot A_{DCan}-K_{tCan}\cdot A_{1Can}$ (Equation S7)

$\frac{dA_{2Can}}{dt}=K_{tCan}\cdot A_{1Can}-K_{tCan}\cdot A_{2Can}$ (Equation S8)

$\frac{dA_{3Can}}{dt}=K_{tCan}\cdot A_{2Can}-K_{tCan}\cdot A_{3Can}$ (Equation S9)

$\frac{dA_{4Can}}{dt}=K_{tCan}\cdot A_{3Can}-K_{tCan}\cdot A_{4Can}$ (Equation S10)

$\frac{dA_{CCan}}{dt}=K_{tCan}\cdot A_{4Can}-\frac{{CL}_{Can}}{V_{CCan}}\cdot A_{CCan}-\frac{{CL}_{DCan}}{V_{CCan}}\cdot A_{CCan}+\frac{{CL}_{DCan}}{V_{TCan}}\cdot A_{TCan}$ (Equation S11)

$\frac{dA_{TCan}}{dt}=\frac{{CL}_{DCan}}{V_{CCan}}\cdot A_{CCan}-\frac{{CL}_{DCan}}{V_{TCan}}\cdot A_{TCan}$ (Equation S12)

Where A_D_ is the drug amount in dosing compartment, that is equal to dose at time zero_._ A_n_ (n=1, 2, 3, 4) is drug amount in transit compartment that is equal to 0 at time zero. A_C_ and A_T_ is drug amount in central and peripheric compartment, respectively, that is equal to 0 at time zero. K_t_ is absorption rate constant as well as transit rate constant. CL is clearance in central compartment. CL_D_ is distribution clearance. V_C_ and V_T_ is central and peripheral compartment volume of distribution, respectively. The subscript character “Can” represents canagliflozin.

1.2 Interstudy variability and residual error model

Interstudy variability of all PK parameters was assumed to be log-normally distributed and was described using exponential model. Combined additive and proportional error model were used to describe residual error. The square root of sample size in each study was used as the weighting of data.

$P_{ij}=\theta_{i}\cdot\exp\left( \eta_{ij} \right)$ (Equation S13)

$Y_{obs}=Y_{pred}+Y_{pred}\cdot\varepsilon_{1}\cdot W+\varepsilon_{2}\cdot W$ (Equation S14)

Where P_ij_ is the estimate of the *i*th parameter in the *j*th study, θ_i_ is the typical values of the *i*th parameter, and η_ij_ quantifies the interstudy variability. Y_obs_ and Y_pred_ are the observed and predicted plasma concentrations. And ε_1_ is the proportional component and ε_2_ is the additive component quantifying the residual error, with both being assumed to be normally distributed in the range from 0 to σ^2^. W is the weight coefficient defining as square root of sample size in each study.

**2. Supplementary Figures**


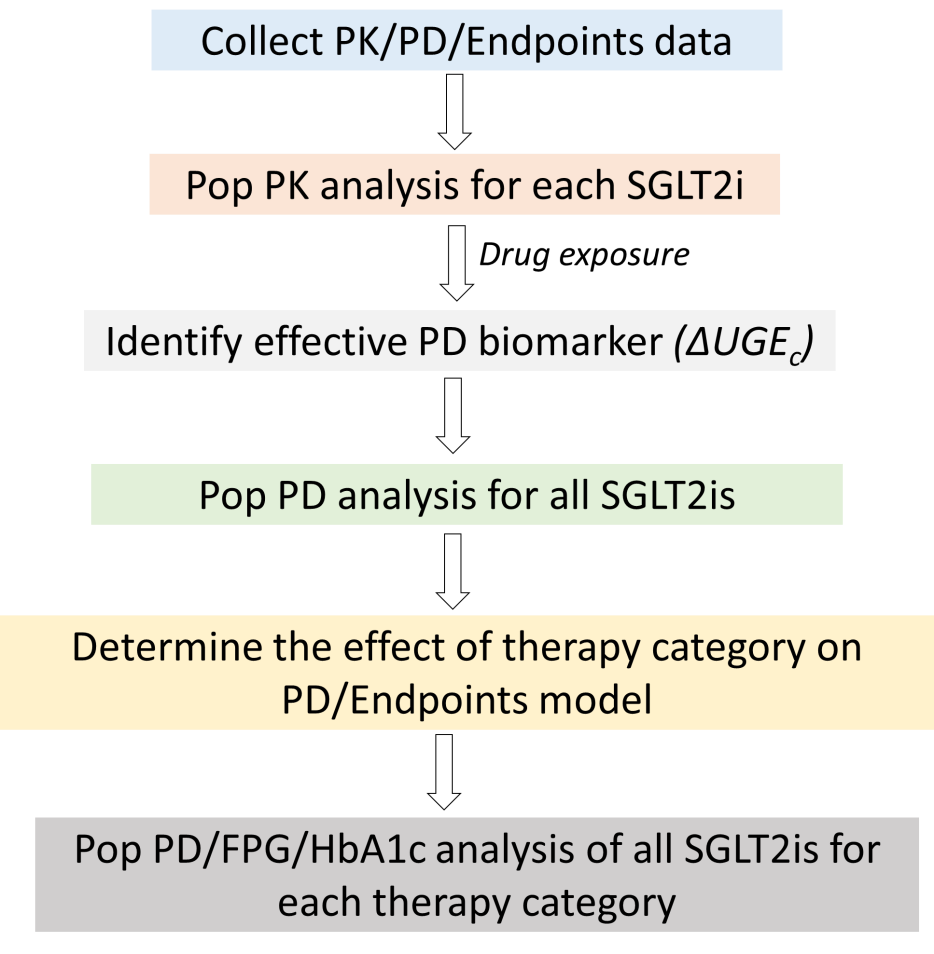


Figure S1. The overall study strategy.


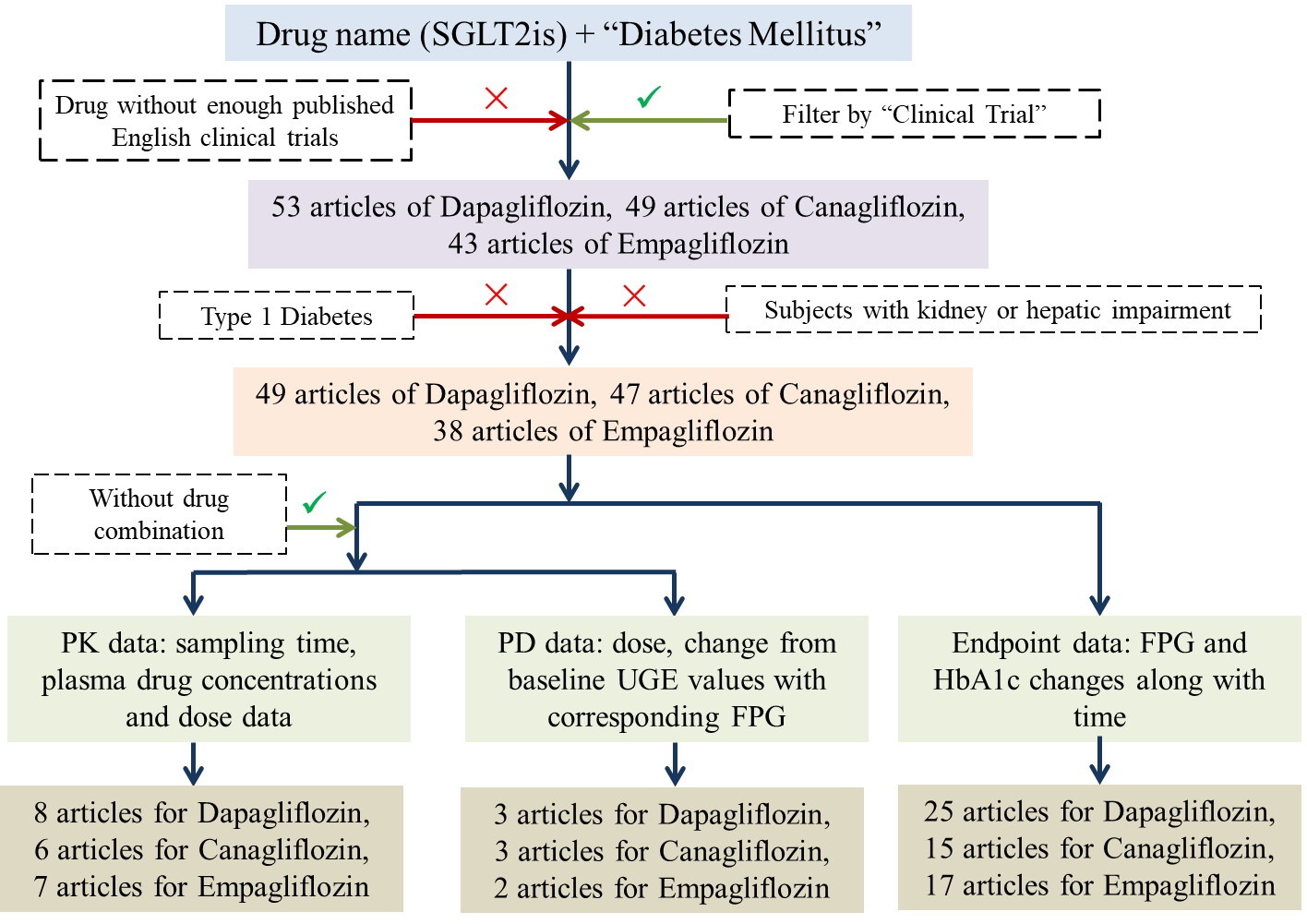


Figure S2. Overview of literature searching strategy in the study. The data is based on the articles published on PubMed before July 2016. The red array with X mark represents the exclusion criteria and the green array with green tick represents the inclusion criteria.


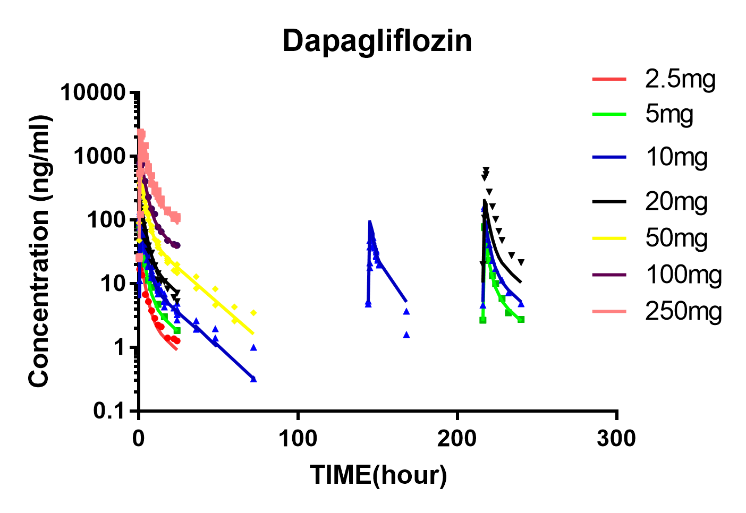


**B**

**A**


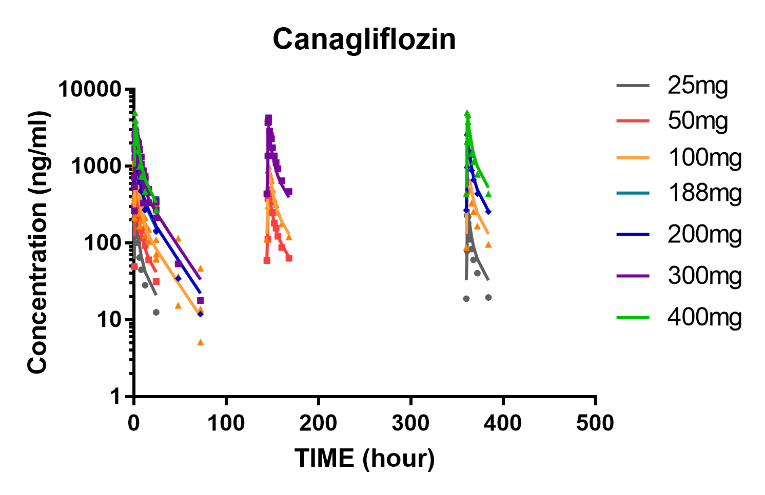


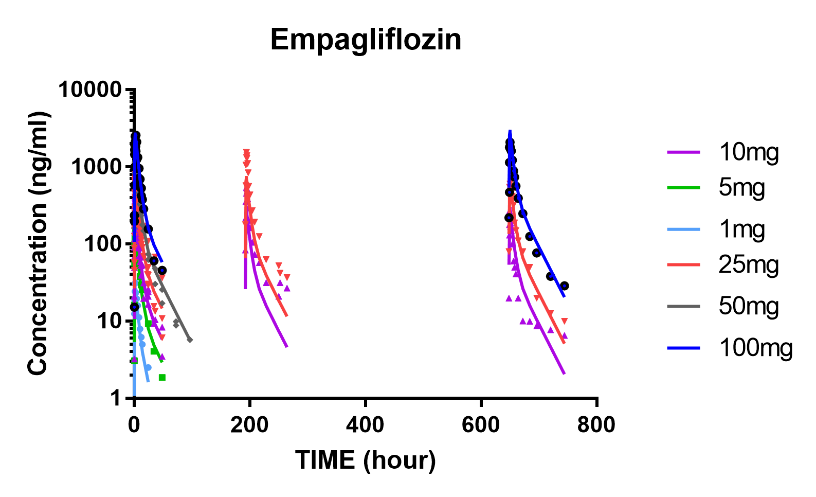


**C**

Figure S3. Semi-log plots of observations and model predictions of PK data of Dapagliflozin (A), Canagliflozin (B) and Empagliflozin (C). The lines are the PK model predictions while the dots are the observations. Different color represents different dosage.


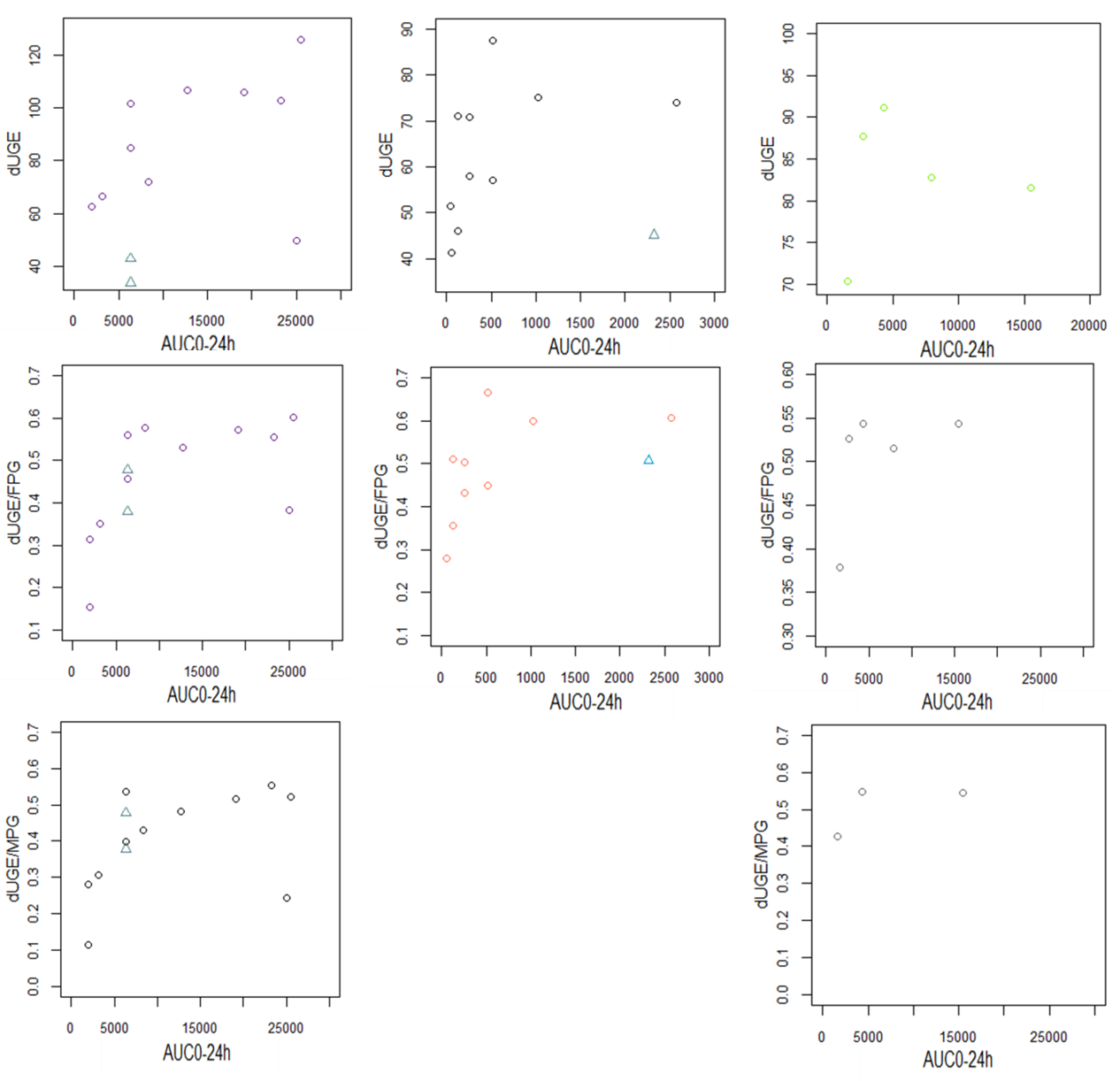


Figure S4. Observed PK (AUC_0-24h_, units: ng/mL∙h ) and biomarker defined as ΔUGE (top panel, units: g), FPG-corrected ΔUGE (middle panel, units: g/(mg/dL)) and MPG-corrected ΔUGE (bottom panel, units: g/(mg/dL)) relationship in Canagliflozin (left), Dapagliflozin (middle) and Empagliflozin (right). The circles represent T2DM patients while triangles represent healthy subjects.

**B**


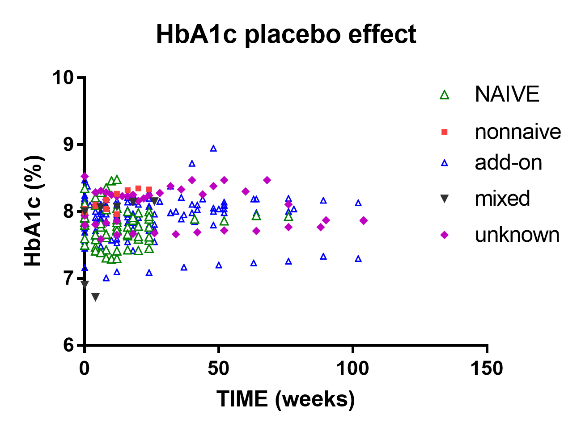

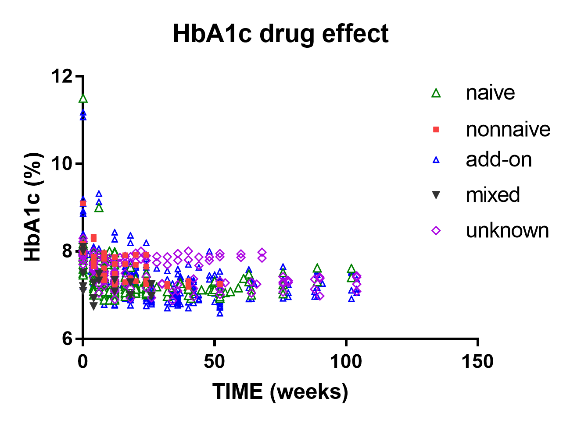

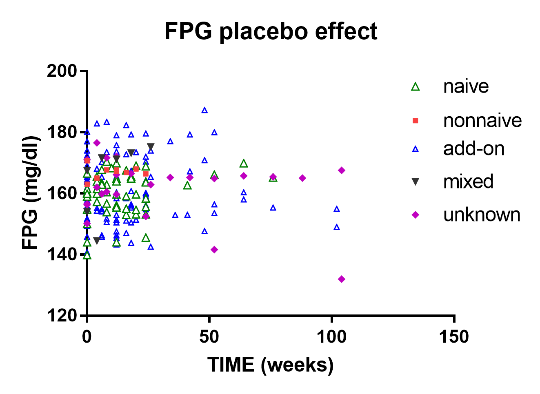

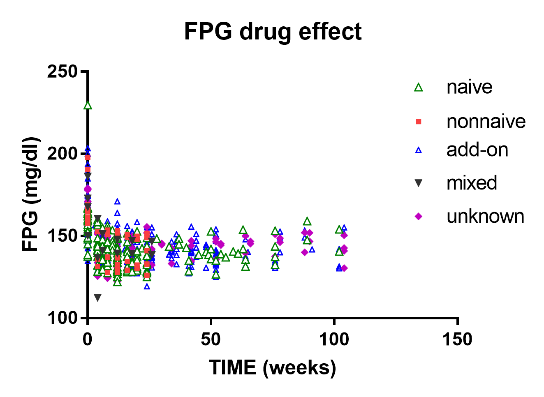


**B**

**A**

**D**

**C**

Figure S5. Observations of endpoints data in HbA1c placebo group (A), HbA1c drug group (B), fasting plasma glucose placebo group (C) and fasting plasma glucose drug effect group (D).

**A**


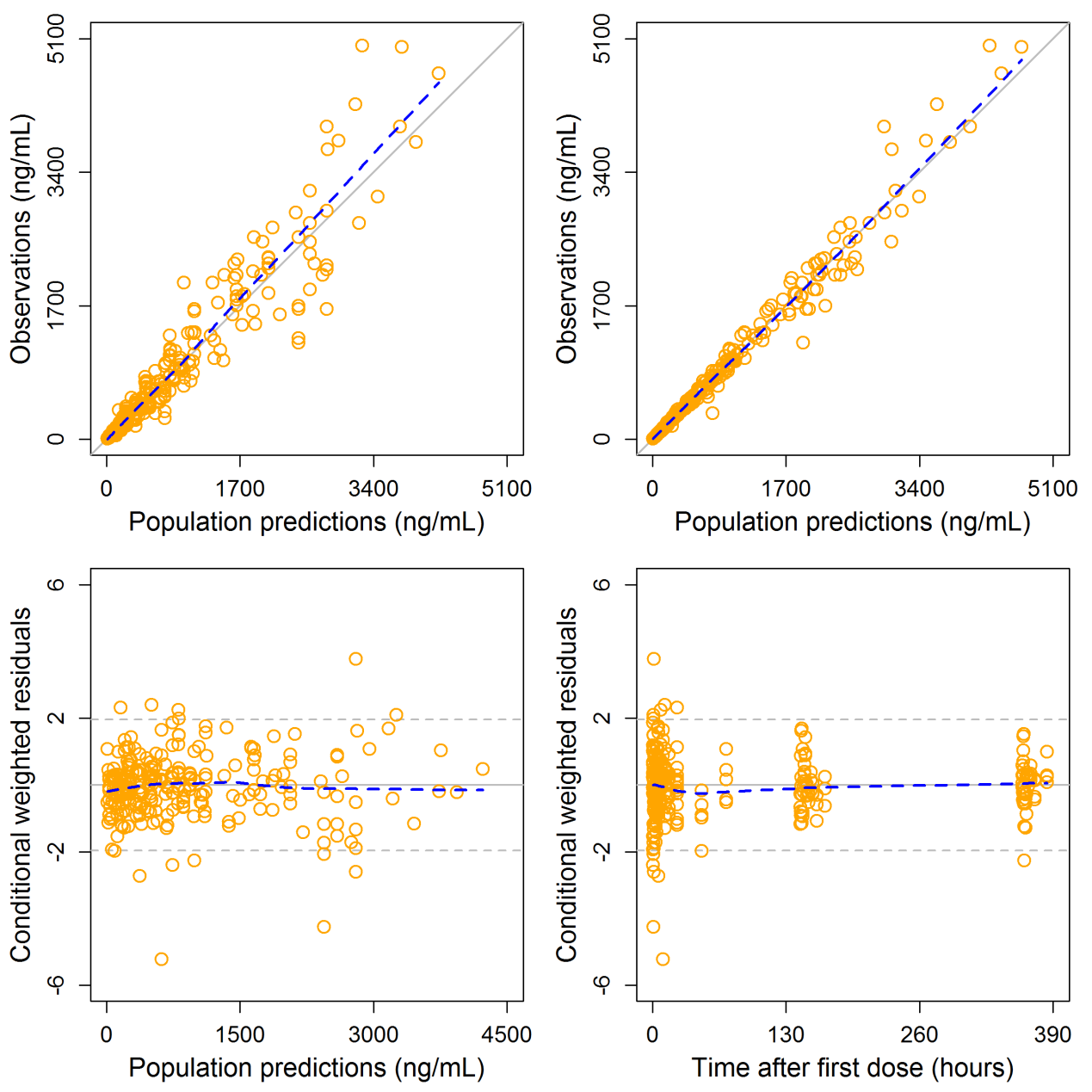


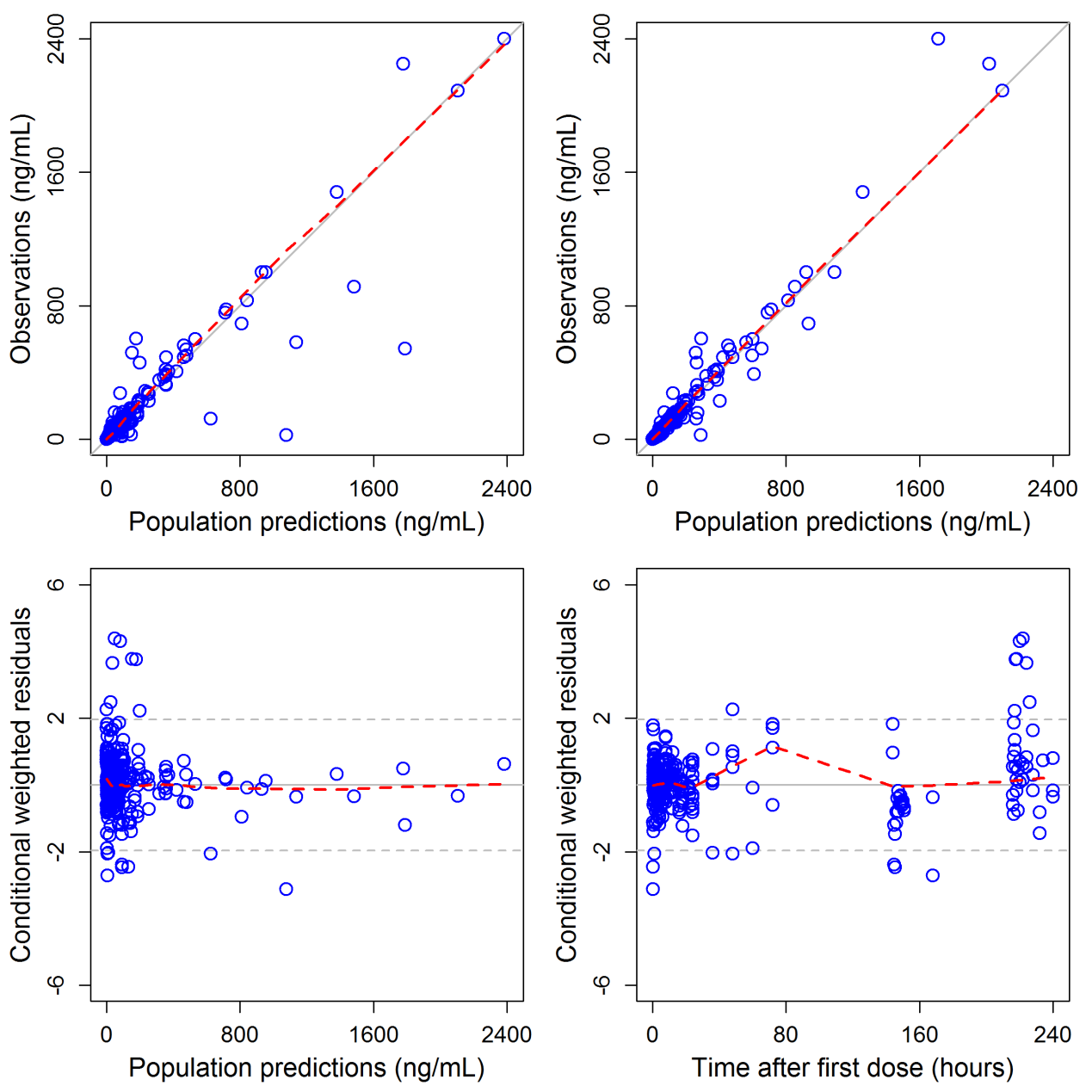


**B**


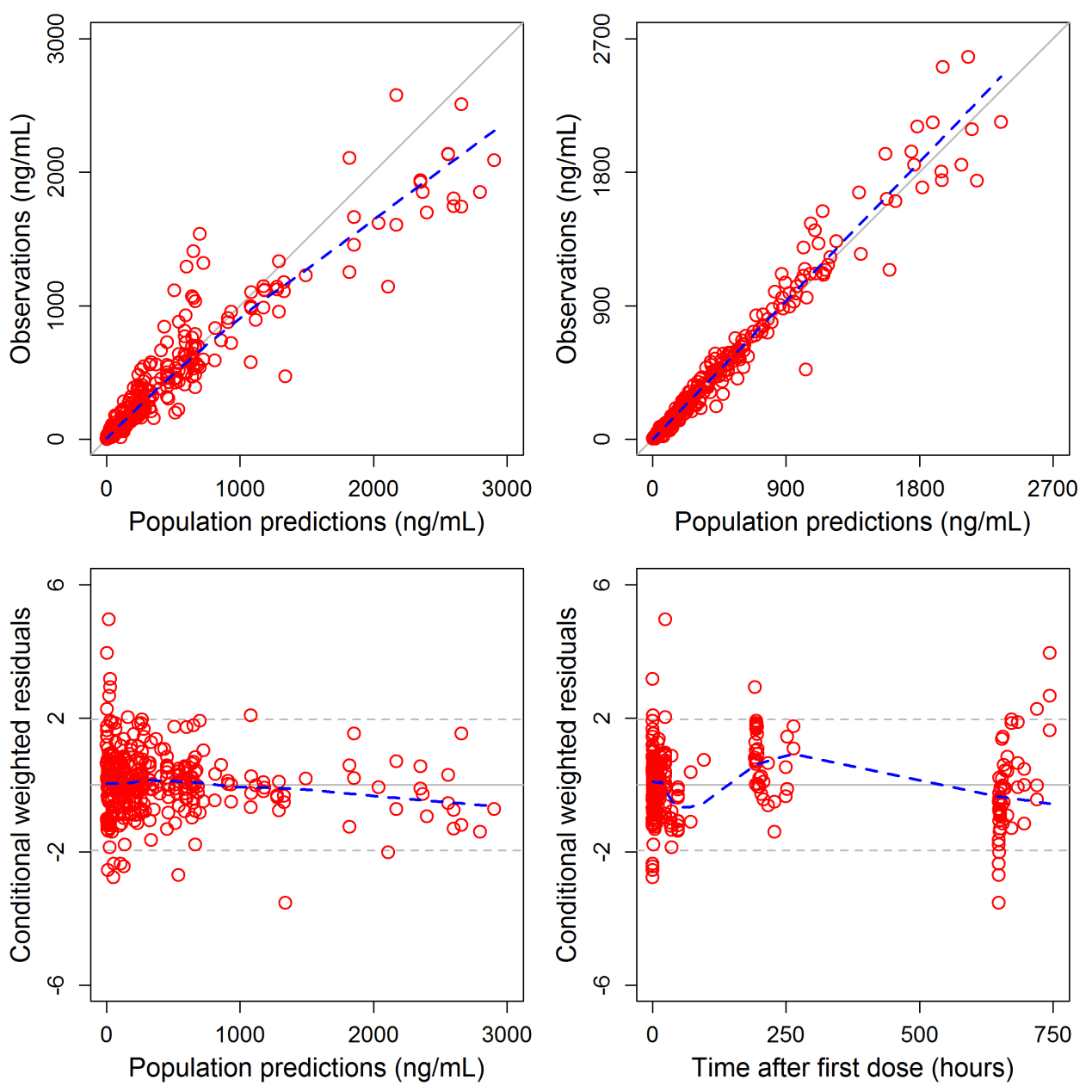


**C**

Figure S6. PK models diagnostic plots of Canagliflozin (A), Dapagliflozin (B) and Empagliflozin (C).


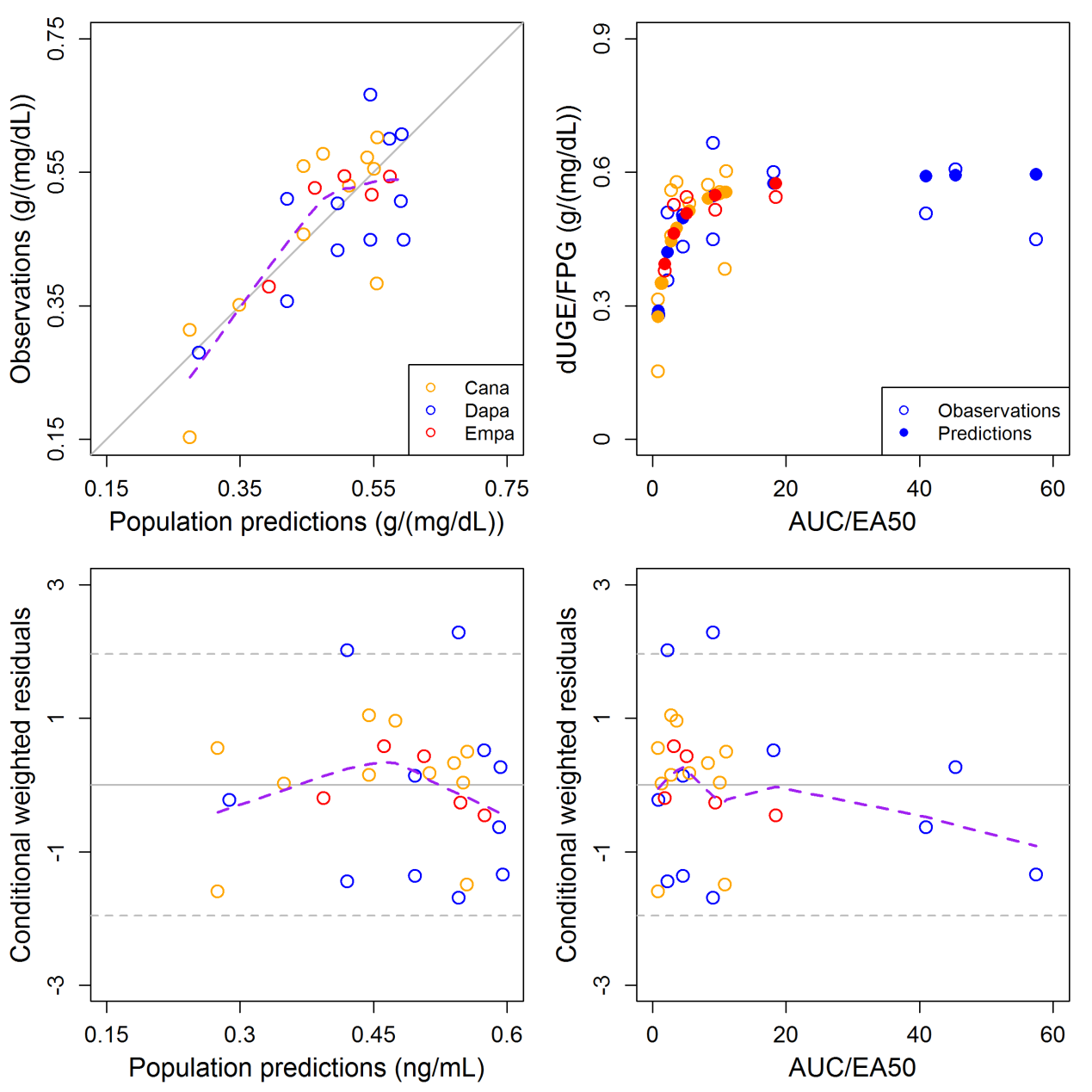


Figure S7. Diagnostic plots of PK/PD models.


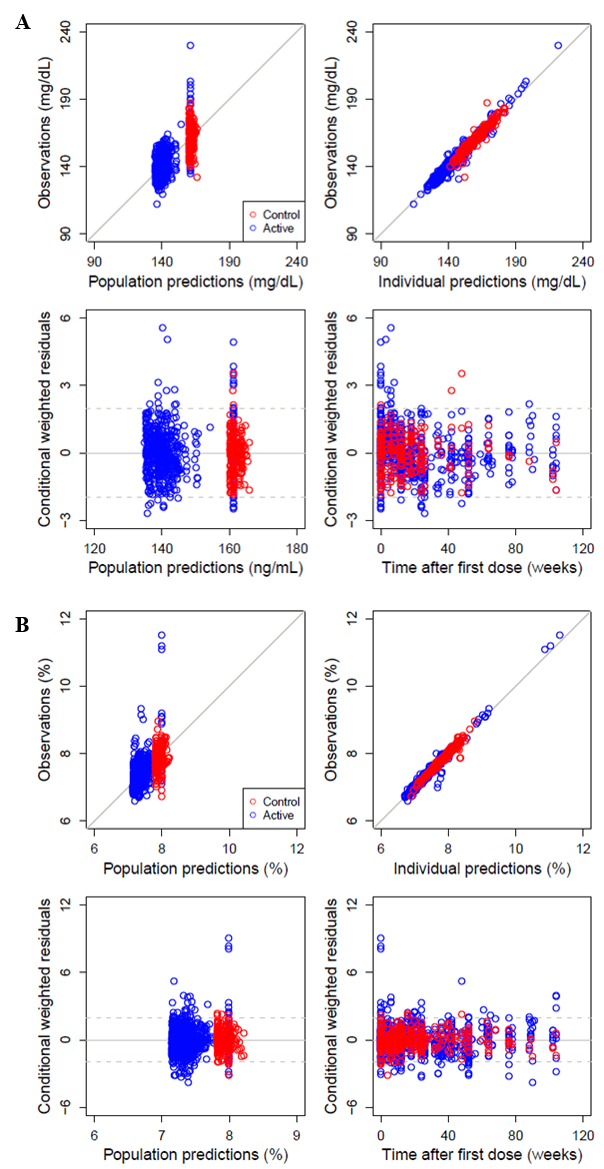


Figure S8. Goodness-of-fit plots of FPG time course (A) and HbA1c time course (B). Red points represent placebo control groups and blue points represent drug treatment groups.

Table S1. Overview of PK studies and exploratory analysis

| SGLT2 inhibitors | Data | Dose (mg) | No. of Subjects | Paper Cited^*^ |
| --- | --- | --- | --- | --- |
| Dapagliflozin | PK | 2.5, 5, 10, 20, 50, 100, 250 | 201 (177 healthy) | [S28-S35] |
|  | PD | 1,2.5,5,10,20,50 | 424 | [S11, S18, S21] |
|  | FPG and HbA1c | 0,1,2.5, 5, 10, 20, 50 | 8324 | [S18, S36-S41, S43-S60] |
| Canagliflozin | PK | 25,50,100,188,200,300,400 | 158 (76 healthy) | [S61-S66] |
|  | PD | 30,50,100,200,300,400 | 137 | [S67-S69] |
|  | FPG and HbA1c | 0,50,100,200,300 | 7004 | [S42, S69-S82] |
| Empagliflozin | PK | 1,5,10,25,50,100  10,25,100  0,1,5,10,12.5,25,50,100 | 204 (122 healthy) | [S83-S89] |
|  | PD  FPG and HbA1c |  | 55 | [S83, S88] |
|  |  |  | 9489 | [S88, S90-S105] |

^*^See the supplementary citation list.

Table S2. Patients demographic baseline. ^a^

| Data | Drug Name | Age (yeas) | BMI  (kg/m^2^) | Body Weight (kg) | Male (%) | FPG Baseline (mg/dl) | UGE baseline (g) | HbA1c baseline (%) |
| --- | --- | --- | --- | --- | --- | --- | --- | --- |
| PK | Dapagliflozin | 38.1  (9.78) | 27.02  (3.3) | 76.38  (14.58) | 74.32  (17.15) | -- | -- | -- |
|  | Canagliflozin | 46.82  (11.15) | 26.44  (3.02) | 75.58  (6.78) | 72.85  (19.18) | -- | -- | -- |
|  | Empagliflozin | 43.52  (15.0) | 24.84  (3.01) | 73.3  (9.18) | 63.16  (27.53) | -- | -- | -- |
| PD | Dapagliflozin | 54.14  (4.82) | -- | -- | 62.21  (13.14) | 150.25  (22.95) | 12.82  (8.6) | -- |
|  | Canagliflozin | 52.19  (3.54) | -- | -- | 60.97  (18.61) | 186.42  (14.1) | 17.42  (10.61) | -- |
|  | Empagliflozin | 56.3  (1.99) | -- | -- | 71.48  (22.08) | 166.19  (13.19) | 6.5  (2.71) | -- |
| Endpoint | Placebo | 57.2  (3.45) | 29.88  (2.78) | 84.65  (9.05) | 56.98  (10.93) | 159.57  (9.47) | -- | 7.93  (0.37) |
|  | Dapagliflozin | 56.08  (4.13) | 30.85  (2.7) | 84.94  (9.02) | 54.57  (10.46) | 162.67  (13.74) | -- | 8.07  (0.44) |
|  | Canagliflozin | 55.8  (1.8) | 29.36  (3.25) | 82.08  (9.87) | 54.95  (10.8) | 163.64  (5.8) | -- | 7.93  (0.17) |
|  | Empagliflozin | 57.57  (3.03) | 28.53  (2.67) | 79.03  (8.57) | 61.39  (11.5) | 161  (16.78) | -- | 8.13  (1.03) |

^a^ mean(sd)

Supplementary paper cited list

1. Ogurtsova, K., et al., *IDF Diabetes Atlas: Global estimates for the prevalence of diabetes for 2015 and 2040.* Diabetes Research and Clinical Practice, 2017. **128**: p. 40-50.

2. Tuomilehto, J., et al., *Epidemiology of macrovascular disease and hypertension in diabetes mellitus.* International textbook of diabetes mellitus, 1997.

3. Ripsin, C.M., H. Kang, and R.J. Urban, *Management of blood glucose in type 2 diabetes mellitus.* Am Fam Physician, 2009. **79**(1): p. 29-36.

4. Inzucchi, S.E., et al., *Management of hyperglycaemia in type 2 diabetes: a patient-centered approach. Position statement of the American Diabetes Association (ADA) and the European Association for the Study of Diabetes (EASD).* Diabetologia, 2012. **55**(6): p. 1577-1596.

5. Marx, N. and D.K. McGuire, *Sodium-glucose cotransporter-2 inhibition for the reduction of cardiovascular events in high-risk patients with diabetes mellitus.* European heart journal, 2016. **37**(42): p. 3192-3200.

6. Madhavi, K., D.S. Reddy, and S. Kulkarni, *SGLT2 Inhibitors: A New Generation of Antidiabetic Drugs.*

7. Nauck, M.A., *Update on developments with SGLT2 inhibitors in the management of type 2 diabetes.* Drug design, development and therapy, 2014. **8**: p. 1335.

8. Scheen, A.J., *Pharmacodynamics, efficacy and safety of sodium–glucose co-transporter type 2 (SGLT2) inhibitors for the treatment of type 2 diabetes mellitus.* Drugs, 2015. **75**(1): p. 33-59.

9. Kim, S.R., et al., *The Relationship between Increases in Morning Spot Urinary Glucose Excretion and Decreases in HbA1C in Patients with Type 2 Diabetes After Taking an SGLT2 Inhibitor: A Retrospective, Longitudinal Study.* Diabetes Therapy, 2017: p. 1-9.

10. Hardman, T.C. and S.W. Dubrey, *Development and potential role of type-2 sodium-glucose transporter inhibitors for management of type 2 diabetes.* Diabetes Therapy, 2011. **2**(3): p. 133-145.

11. Sha, S., et al., *Pharmacodynamic differences between canagliflozin and dapagliflozin: results of a randomized, double‐blind, crossover study.* Diabetes, Obesity and Metabolism, 2015. **17**(2): p. 188-197.

12. Seman, L., et al., *Empagliflozin (BI 10773), a potent and selective SGLT2 inhibitor, induces dose‐dependent glucosuria in healthy subjects.* Clinical pharmacology in drug development, 2013. **2**(2): p. 152-161.

13. Riggs, M.M., et al., *Exposure− response modelling for empagliflozin, a sodium glucose cotransporter 2 (SGLT2) inhibitor, in patients with type 2 diabetes.* British journal of clinical pharmacology, 2014. **78**(6): p. 1407-1418.

14. Stringer, F., et al., *Evaluation of the long-term durability and glycemic control of fasting plasma glucose and glycosylated hemoglobin for pioglitazone in Japanese patients with type 2 diabetes.* Diabetes technology & therapeutics, 2015. **17**(3): p. 215-223.

15. Samtani, M.N., *Simple pharmacometric tools for oral anti‐diabetic drug development: competitive landscape for oral non‐insulin therapies in type 2 diabetes.* Biopharmaceutics & drug disposition, 2010. **31**(2‐3): p. 162-177.

16. Holford, N., *An introduction to visual predictive checks*. 2012.

17. Savic, R.M., et al., *Implementation of a transit compartment model for describing drug absorption in pharmacokinetic studies.* Journal of pharmacokinetics and pharmacodynamics, 2007. **34**(5): p. 711-726.

18. Kasichayanula, S., et al., *Pharmacokinetics and pharmacodynamics of dapagliflozin, a novel selective inhibitor of sodium–glucose co‐transporter type 2, in Japanese subjects without and with type 2 diabetes mellitus.* Diabetes, Obesity and Metabolism, 2011. **13**(4): p. 357-365.

19. Plosker, G.L., *Canagliflozin: a review of its use in patients with type 2 diabetes mellitus.* Drugs, 2014. **74**(7): p. 807-824.

20. Scheen, A.J., *Pharmacokinetic and pharmacodynamic profile of empagliflozin, a sodium glucose co-transporter 2 inhibitor.* Clinical pharmacokinetics, 2014. **53**(3): p. 213-225.

21. Komoroski, B., et al., *Dapagliflozin, a novel, selective SGLT2 inhibitor, improved glycemic control over 2 weeks in patients with type 2 diabetes mellitus.* Clinical Pharmacology & Therapeutics, 2009. **85**(5): p. 513-519.

22. Kang, S.-Y., M.-J. Kim, and J.-H. Lee, *1, 2, 4-Triazinylmethylphenyl glucoside as novel C-aryl glucoside SGLT2 inhibitors.* Bulletin of the Korean Chemical Society, 2011. **32**(spc8): p. 2938-2940.

23. Villani, L., *Canagliflozin Potently Activates AMPK and Inhibits the Growth and Survival of Cancer Cells.* 2015.

24. Hu, J., et al., *Empagliflozin/metformin fixed-dose combination: a review in patients with type 2 diabetes.* Expert opinion on pharmacotherapy, 2016. **17**(18): p. 2471-2477.

25. Demin Jr, O., et al., *Analysis of the efficacy of SGLT2 inhibitors using semi-mechanistic model.* Frontiers in pharmacology, 2014. **5**.

26. Ozmen, S., et al., *A simple way to estimate mean plasma glucose and to identify type 2 diabetic subjects with poor glycaemic control when a standardized HbA1c assay is not available.* Diabetic medicine, 2006. **23**(10): p. 1151-1154.

27. Monnier, L., H. Lapinski, and C. Colette, *Contributions of fasting and postprandial plasma glucose increments to the overall diurnal hyperglycemia of type 2 diabetic patients.* Diabetes care, 2003. **26**(3): p. 881-885.

28. Yang, L., et al., *Pharmacokinetic and pharmacodynamic properties of single-and multiple-dose of dapagliflozin, a selective inhibitor of SGLT2, in healthy Chinese subjects.* Clinical therapeutics, 2013. **35**(8): p. 1211-1222. e2.

29. DeFronzo, R.A., et al., *Characterization of renal glucose reabsorption in response to dapagliflozin in healthy subjects and subjects with type 2 diabetes.* Diabetes Care, 2013. **36**(10): p. 3169-3176.

30. Kasichayanula, S., et al., *The influence of kidney function on dapagliflozin exposure, metabolism and pharmacodynamics in healthy subjects and in patients with type 2 diabetes mellitus.* British journal of clinical pharmacology, 2013. **76**(3): p. 432-444.

31. Kasichayanula, S., et al., *Effects of rifampin and mefenamic acid on the pharmacokinetics and pharmacodynamics of dapagliflozin.* Diabetes, Obesity and Metabolism, 2013. **15**(3): p. 280-283.

32. Kasichayanula, S., et al., *Lack of pharmacokinetic interactions between dapagliflozin and simvastatin, valsartan, warfarin, or digoxin.* Advances in therapy, 2012. **29**(2): p. 163-177.

33. Kasichayanula, S., et al., *Influence of hepatic impairment on the pharmacokinetics and safety profile of dapagliflozin: an open-label, parallel-group, single-dose study.* Clinical therapeutics, 2011. **33**(11): p. 1798-1808.

34. Kasichayanula, S., et al., *Effect of a high‐fat meal on the pharmacokinetics of dapagliflozin, a selective SGLT2 inhibitor, in healthy subjects.* Diabetes, Obesity and Metabolism, 2011. **13**(8): p. 770-773.

35. Komoroski, B., et al., *Dapagliflozin, a novel SGLT2 inhibitor, induces dose‐dependent glucosuria in healthy subjects.* Clinical Pharmacology & Therapeutics, 2009. **85**(5): p. 520-526.

36. Kaku, K., et al., *Efficacy and safety of dapagliflozin as a monotherapy for type 2 diabetes mellitus in Japanese patients with inadequate glycaemic control: a phase II multicentre, randomized, double‐blind, placebo‐controlled trial.* Diabetes, Obesity and Metabolism, 2013. **15**(5): p. 432-440.

37. List, J.F., et al., *Sodium-glucose cotransport inhibition with dapagliflozin in type 2 diabetes.* Diabetes care, 2009. **32**(4): p. 650-657.

38. Matthaei, S., et al., *Dapagliflozin improves glycemic control and reduces body weight as add-on therapy to metformin plus sulfonylurea: a 24-week randomized, double-blind clinical trial.* Diabetes Care, 2015. **38**(3): p. 365-372.

39. Bailey, C., et al., *Efficacy and safety of dapagliflozin monotherapy in people with type 2 diabetes: a randomized double‐blind placebo‐controlled 102‐week trial.* Diabetic Medicine, 2015. **32**(4): p. 531-541.

40. Rosenstock, J., et al., *Dual add-on therapy in type 2 diabetes poorly controlled with metformin monotherapy: a randomized double-blind trial of saxagliptin plus dapagliflozin addition versus single addition of saxagliptin or dapagliflozin to metformin.* Diabetes Care, 2015. **38**(3): p. 376-383.

41. Schumm‐Draeger, P.M., et al., *Twice‐daily dapagliflozin co‐administered with metformin in type 2 diabetes: a 16‐week randomized, placebo‐controlled clinical trial.* Diabetes, Obesity and Metabolism, 2015. **17**(1): p. 42-51.

42. Inagaki, N., et al., *Efficacy and safety of canagliflozin monotherapy in Japanese patients with type 2 diabetes inadequately controlled with diet and exercise: a 24-week, randomized, double-blind, placebo-controlled, Phase III study.* Expert opinion on pharmacotherapy, 2014. **15**(11): p. 1501-1515.

43. Kaku, K., et al., *Efficacy and safety of dapagliflozin monotherapy in Japanese patients with type 2 diabetes inadequately controlled by diet and exercise.* Diabetes, Obesity and Metabolism, 2014. **16**(11): p. 1102-1110.

44. Leiter, L.A., et al., *Dapagliflozin added to usual care in individuals with type 2 diabetes mellitus with preexisting cardiovascular disease: a 24‐week, multicenter, randomized, double‐blind, placebo‐controlled study with a 28‐week extension.* Journal of the American Geriatrics Society, 2014. **62**(7): p. 1252-1262.

45. Ji, L., et al., *Dapagliflozin as monotherapy in drug-naive Asian patients with type 2 diabetes mellitus: a randomized, blinded, prospective phase III study.* Clinical therapeutics, 2014. **36**(1): p. 84-100. e9.

46. Mudaliar, S., et al., *Changes in insulin sensitivity and insulin secretion with the sodium glucose cotransporter 2 inhibitor dapagliflozin.* Diabetes technology & therapeutics, 2014. **16**(3): p. 137-144.

47. Jabbour, S.A., et al., *Dapagliflozin is effective as add-on therapy to sitagliptin with or without metformin: a 24-week, multicenter, randomized, double-blind, placebo-controlled study.* Diabetes Care, 2014. **37**(3): p. 740-750.

48. Kohan, D.E., et al., *Long-term study of patients with type 2 diabetes and moderate renal impairment shows that dapagliflozin reduces weight and blood pressure but does not improve glycemic control.* Kidney international, 2014. **85**(4): p. 962-971.

49. Wilding, J., et al., *Dapagliflozin in patients with type 2 diabetes receiving high doses of insulin: efficacy and safety over 2 years.* Diabetes, Obesity and Metabolism, 2014. **16**(2): p. 124-136.

50. Bolinder, J., et al., *Dapagliflozin maintains glycaemic control while reducing weight and body fat mass over 2 years in patients with type 2 diabetes mellitus inadequately controlled on metformin.* Diabetes, Obesity and Metabolism, 2014. **16**(2): p. 159-169.

51. Lambers Heerspink, H., et al., *Dapagliflozin a glucose‐regulating drug with diuretic properties in subjects with type 2 diabetes.* Diabetes, Obesity and Metabolism, 2013. **15**(9): p. 853-862.

52. Bailey, C.J., et al., *Dapagliflozin add-on to metformin in type 2 diabetes inadequately controlled with metformin: a randomized, double-blind, placebo-controlled 102-week trial.* BMC medicine, 2013. **11**(1): p. 43.

53. Bailey, C., et al., *Dapagliflozin monotherapy in drug‐naïve patients with diabetes: a randomized‐controlled trial of low‐dose range.* Diabetes, Obesity and Metabolism, 2012. **14**(10): p. 951-959.

54. Rosenstock, J., et al., *Effects of dapagliflozin, an SGLT2 inhibitor, on HbA1c, body weight, and hypoglycemia risk in patients with type 2 diabetes inadequately controlled on pioglitazone monotherapy.* Diabetes care, 2012. **35**(7): p. 1473-1478.

55. Henry, R., et al., *Dapagliflozin, metformin XR, or both: initial pharmacotherapy for type 2 diabetes, a randomised controlled trial.* International journal of clinical practice, 2012. **66**(5): p. 446-456.

56. Strojek, K., et al., *Effect of dapagliflozin in patients with type 2 diabetes who have inadequate glycaemic control with glimepiride: a randomized, 24‐week, double‐blind, placebo‐controlled trial.* Diabetes, Obesity and Metabolism, 2011. **13**(10): p. 928-938.

57. Ferrannini, E., et al., *Dapagliflozin monotherapy in type 2 diabetic patients with inadequate glycemic control by diet and exercise.* Diabetes care, 2010. **33**(10): p. 2217-2224.

58. Zhang, L., et al., *Dapagliflozin treatment in patients with different stages of type 2 diabetes mellitus: effects on glycaemic control and body weight.* Diabetes, Obesity and Metabolism, 2010. **12**(6): p. 510-516.

59. Matthaei, S., et al., *Randomized, double-blind trial of triple therapy with saxagliptin add-on to dapagliflozin plus metformin in patients with type 2 diabetes.* Diabetes Care, 2015. **38**(11): p. 2018-2024.

60. Mathieu, C., et al., *Randomized, double-blind, phase 3 trial of triple therapy with dapagliflozin add-on to saxagliptin plus metformin in type 2 diabetes.* Diabetes Care, 2015. **38**(11): p. 2009-2017.

61. Iijima, H., et al., *Pharmacokinetics, pharmacodynamics, and safety of canagliflozin in Japanese patients with type 2 diabetes mellitus.* Advances in therapy, 2015. **32**(8): p. 768-782.

62. Chen, X., et al., *Pharmacokinetics, pharmacodynamics, and safety of single-dose canagliflozin in healthy Chinese subjects.* Clinical therapeutics, 2015. **37**(7): p. 1483-1492. e1.

63. Devineni, D., et al., *Effects of rifampin, cyclosporine A, and probenecid on the pharmacokinetic profile of canagliflozin, a sodium glucose co-transporter 2 inhibitor, in healthy participants.* International journal of clinical pharmacology and therapeutics, 2015. **53**(2): p. 115.

64. Inagaki, N., et al., *Pharmacokinetic and pharmacodynamic profiles of canagliflozin in Japanese patients with type 2 diabetes mellitus and moderate renal impairment.* Clinical drug investigation, 2014. **34**(10): p. 731-742.

65. Mamidi, R.N., et al., *Metabolism and excretion of canagliflozin in mice, rats, dogs, and humans.* Drug Metabolism and Disposition, 2014. **42**(5): p. 903-916.

66. Devineni, D., et al., *Pharmacokinetics and pharmacodynamics of canagliflozin, a sodium glucose co‐transporter 2 inhibitor, in subjects with type 2 diabetes mellitus.* The Journal of Clinical Pharmacology, 2013. **53**(6): p. 601-610.

67. Sha, S., et al., *Pharmacodynamic effects of canagliflozin, a sodium glucose co-transporter 2 inhibitor, from a randomized study in patients with type 2 diabetes.* PLoS One, 2014. **9**(8): p. e105638.

68. Devineni, D., et al., *Canagliflozin improves glycaemic control over 28 days in subjects with type 2 diabetes not optimally controlled on insulin.* Diabetes, Obesity and Metabolism, 2012. **14**(6): p. 539-545.

69. Inagaki, N., et al., *Effects of baseline blood pressure and low-density lipoprotein cholesterol on safety and efficacy of canagliflozin in Japanese patients with type 2 diabetes mellitus.* Advances in therapy, 2015. **32**(11): p. 1085-1103.

70. Bode, B., et al., *Long‐term efficacy and safety of canagliflozin over 104 weeks in patients aged 55–80 years with type 2 diabetes.* Diabetes, Obesity and Metabolism, 2015. **17**(3): p. 294-303.

71. Leiter, L.A., et al., *Canagliflozin provides durable glycemic improvements and body weight reduction over 104 weeks versus glimepiride in patients with type 2 diabetes on metformin: a randomized, double-blind, phase 3 study.* Diabetes Care, 2015. **38**(3): p. 355-364.

72. Ji, L., et al., *Canagliflozin in Asian patients with type 2 diabetes on metformin alone or metformin in combination with sulphonylurea.* Diabetes, Obesity and Metabolism, 2015. **17**(1): p. 23-31.

73. Polidori, D., A. Mari, and E. Ferrannini, *Canagliflozin, a sodium glucose co-transporter 2 inhibitor, improves model-based indices of beta cell function in patients with type 2 diabetes.* Diabetologia, 2014. **57**(5): p. 891-901.

74. Forst, T., et al., *Efficacy and safety of canagliflozin over 52 weeks in patients with type 2 diabetes on background metformin and pioglitazone.* Diabetes, Obesity and Metabolism, 2014. **16**(5): p. 467-477.

75. Wilding, J., et al., *Efficacy and safety of canagliflozin in patients with type 2 diabetes mellitus inadequately controlled with metformin and sulphonylurea: a randomised trial.* International journal of clinical practice, 2013. **67**(12): p. 1267-1282.

76. Lavalle-González, F., et al., *Efficacy and safety of canagliflozin compared with placebo and sitagliptin in patients with type 2 diabetes on background metformin monotherapy: a randomised trial.* Diabetologia, 2013. **56**(12): p. 2582-2592.

77. Cefalu, W.T., et al., *Efficacy and safety of canagliflozin versus glimepiride in patients with type 2 diabetes inadequately controlled with metformin (CANTATA-SU): 52 week results from a randomised, double-blind, phase 3 non-inferiority trial.* The Lancet, 2013. **382**(9896): p. 941-950.

78. Inagaki, N., et al., *Efficacy and safety of canagliflozin in Japanese patients with type 2 diabetes: a randomized, double‐blind, placebo‐controlled, 12‐week study.* Diabetes, Obesity and Metabolism, 2013. **15**(12): p. 1136-1145.

79. Schernthaner, G., et al., *Canagliflozin compared with sitagliptin for patients with type 2 diabetes who do not have adequate glycemic control with metformin plus sulfonylurea.* Diabetes care, 2013. **36**(9): p. 2508-2515.

80. Stenlöf, K., et al., *Efficacy and safety of canagliflozin monotherapy in subjects with type 2 diabetes mellitus inadequately controlled with diet and exercise.* Diabetes, Obesity and Metabolism, 2013. **15**(4): p. 372-382.

81. Rosenstock, J., et al., *Dose-ranging effects of canagliflozin, a sodium-glucose cotransporter 2 inhibitor, as add-on to metformin in subjects with type 2 diabetes.* Diabetes care, 2012. **35**(6): p. 1232-1238.

82. Stenlöf, K., et al., *Long-term efficacy and safety of canagliflozin monotherapy in patients with type 2 diabetes inadequately controlled with diet and exercise: findings from the 52-week CANTATA-M study.* Current medical research and opinion, 2014. **30**(2): p. 163-175.

83. Zhao, X., et al., *Pharmacokinetic and pharmacodynamic properties and tolerability of single-and multiple-dose once-daily empagliflozin, a sodium glucose cotransporter 2 inhibitor, in Chinese patients with type 2 diabetes mellitus.* Clinical therapeutics, 2015. **37**(7): p. 1493-1502.

84. Macha, S., et al., *Effect of gemfibrozil, rifampicin, or probenecid on the pharmacokinetics of the SGLT2 inhibitor empagliflozin in healthy volunteers.* Clinical therapeutics, 2014. **36**(2): p. 280-290. e1.

85. Macha, S., et al., *Pharmacokinetics, safety and tolerability of empagliflozin, a sodium glucose cotransporter 2 inhibitor, in patients with hepatic impairment.* Diabetes, Obesity and Metabolism, 2014. **16**(2): p. 118-123.

86. Macha, S., et al., *Pharmacokinetics, pharmacodynamics and safety of empagliflozin, a sodium glucose cotransporter 2 (SGLT2) inhibitor, in subjects with renal impairment.* Diabetes, Obesity and Metabolism, 2014. **16**(3): p. 215-222.

87. Macha, S., et al., *Lack of clinically relevant drug–drug interaction between empagliflozin, a sodium glucose cotransporter 2 inhibitor, and verapamil, ramipril, or digoxin in healthy volunteers.* Clinical therapeutics, 2013. **35**(3): p. 226-235.

88. Heise, T., et al., *Safety, tolerability, pharmacokinetics and pharmacodynamics following 4 weeks' treatment with empagliflozin once daily in patients with type 2 diabetes.* Diabetes, Obesity and Metabolism, 2013. **15**(7): p. 613-621.

89. Sarashina, A., et al., *Safety, tolerability, pharmacokinetics and pharmacodynamics of single doses of empagliflozin, a sodium glucose cotransporter 2 (SGLT2) inhibitor, in healthy Japanese subjects.* Drug metabolism and pharmacokinetics, 2013. **28**(3): p. 213-219.

90. Roden, M., et al., *Safety, tolerability and effects on cardiometabolic risk factors of empagliflozin monotherapy in drug-naïve patients with type 2 diabetes: a double-blind extension of a Phase III randomized controlled trial.* Cardiovascular diabetology, 2015. **14**(1): p. 154.

91. Haering, H.-U., et al., *Empagliflozin as add-on to metformin plus sulphonylurea in patients with type 2 diabetes.* Diabetes research and clinical practice, 2015. **110**(1): p. 82-90.

92. Kovacs, C.S., et al., *Empagliflozin as add-on therapy to pioglitazone with or without metformin in patients with type 2 diabetes mellitus.* Clinical therapeutics, 2015. **37**(8): p. 1773-1788. e1.

93. Ross, S., et al., *Efficacy and safety of empagliflozin twice daily versus once daily in patients with type 2 diabetes inadequately controlled on metformin: a 16‐week, randomized, placebo‐controlled trial.* Diabetes, Obesity and Metabolism, 2015. **17**(7): p. 699-702.

94. Araki, E., et al., *Long‐term treatment with empagliflozin as add‐on to oral antidiabetes therapy in Japanese patients with type 2 diabetes mellitus.* Diabetes, Obesity and Metabolism, 2015. **17**(7): p. 665-674.

95. Lewin, A., et al., *Initial combination of empagliflozin and linagliptin in subjects with type 2 diabetes.* Diabetes care, 2015. **38**(3): p. 394-402.

96. DeFronzo, R.A., et al., *Combination of empagliflozin and linagliptin as second-line therapy in subjects with type 2 diabetes inadequately controlled on metformin.* Diabetes Care, 2015. **38**(3): p. 384-393.

97. Tikkanen, I., et al., *Empagliflozin reduces blood pressure in patients with type 2 diabetes and hypertension.* Diabetes Care, 2015. **38**(3): p. 420-428.

98. Häring, H.-U., et al., *Empagliflozin as add-on to metformin in patients with type 2 diabetes: a 24-week, randomized, double-blind, placebo-controlled trial.* Diabetes care, 2014. **37**(6): p. 1650-1659.

99. Ferrannini, E., et al., *Long-term safety and efficacy of empagliflozin, sitagliptin, and metformin.* Diabetes care, 2013. **36**(12): p. 4015-4021.

100. Häring, H.-U., et al., *Empagliflozin as add-on to metformin plus sulfonylurea in patients with type 2 diabetes.* Diabetes care, 2013. **36**(11): p. 3396-3404.

101. Kovacs, C., et al., *Empagliflozin improves glycaemic and weight control as add‐on therapy to pioglitazone or pioglitazone plus metformin in patients with type 2 diabetes: a 24‐week, randomized, placebo‐controlled trial.* Diabetes, Obesity and Metabolism, 2014. **16**(2): p. 147-158.

102. Rosenstock, J., et al., *Efficacy and safety of empagliflozin, a sodium glucose cotransporter 2 (SGLT2) inhibitor, as add‐on to metformin in type 2 diabetes with mild hyperglycaemia.* Diabetes, Obesity and Metabolism, 2013. **15**(12): p. 1154-1160.

103. Ferrannini, E., et al., *A phase IIb, randomized, placebo‐controlled study of the SGLT2 inhibitor empagliflozin in patients with type 2 diabetes.* Diabetes, Obesity and Metabolism, 2013. **15**(8): p. 721-728.

104. Kadowaki, T., et al., *Empagliflozin monotherapy in Japanese patients with type 2 diabetes mellitus: a randomized, 12-week, double-blind, placebo-controlled, phase II trial.* Advances in therapy, 2014. **31**(6): p. 621-638.

105. Roden, M., et al., *Empagliflozin monotherapy with sitagliptin as an active comparator in patients with type 2 diabetes: a randomised, double-blind, placebo-controlled, phase 3 trial.* The Lancet Diabetes & Endocrinology, 2013. **1**(3): p. 208-219.
